# Digital Electrochemistry for On‐Chip Heterogeneous Material Integration

**DOI:** 10.1002/adma.202101272

**Published:** 2021-05-24

**Authors:** Bin Bao, Boris Rivkin, Farzin Akbar, Dmitriy D. Karnaushenko, Vineeth Kumar Bandari, Laura Teuerle, Christian Becker, Stefan Baunack, Daniil Karnaushenko, Oliver G. Schmidt

**Affiliations:** ^1^ Institute for Integrative Nanosciences Leibniz IFW Dresden 01069 Dresden Germany; ^2^ Material Systems for Nanoelectronics Chemnitz University of Technology 09107 Chemnitz Germany; ^3^ Center for Materials, Architectures and Integration of Nanomembranes (MAIN) Chemnitz University of Technology 09126 Chemnitz Germany; ^4^ Nanophysics, Faculty of Physics TU Dresden 01062 Dresden Germany

**Keywords:** electrochemical actuators, electrochemical depositions, electrochromic displays, heterogeneous integration, indium‐gallium‐zinc oxide active matrices

## Abstract

Many modern electronic applications rely on functional units arranged in an active‐matrix integrated on a single chip. The active‐matrix allows numerous identical device pixels to be addressed within a single system. However, next‐generation electronics requires heterogeneous integration of dissimilar devices, where sensors, actuators, and display pixels sense and interact with the local environment. Heterogeneous material integration allows the reduction of size, increase of functionality, and enhancement of performance; however, it is challenging since front‐end fabrication technologies in microelectronics put extremely high demands on materials, fabrication protocols, and processing environments. To overcome the obstacle in heterogeneous material integration, digital electrochemistry is explored here, which site‐selectively carries out electrochemical processes to deposit and address electroactive materials within the pixel array. More specifically, an amorphous indium‐gallium‐zinc oxide (a‐IGZO) thin‐film‐transistor (TFT) active‐matrix is used to address pixels within the matrix and locally control electrochemical reactions for material growth and actuation. The digital electrochemistry procedure is studied in‐depth by using polypyrrole (PPy) as a model material. Active‐matrix‐driven multicolored electrochromic patterns and actuator arrays are fabricated to demonstrate the capabilities of this approach for material integration. The approach can be extended to a broad range of materials and structures, opening up a new path for advanced heterogeneous microsystem integration.

## Graphical abstract

A novel digital electrochemistry approach is proposed, which site‐selectively carries out electrochemical reactions to deposit electroactive materials. Multicolored electrochromic patterns and actuator arrays are fabricated. The approach can be extended to a broad range of materials and structures, opening up a new path for advanced heterogeneous microsystem integration.

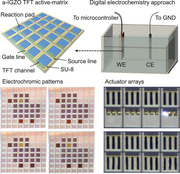

## 1 Introduction

Electrochemistry sits at the heart of vital functionalities for energy storage and generation, electrochemical sensing and actuation.^[^ ^1^ ^]^ Electrochemical devices like batteries, electrochromic displays, electrochemical biosensors, and actuators play critical roles in power supplies, consumer electronics, and healthcare monitoring.^[^ ^2^ ^]^ Various electrochemical sensors and displays have already been merged with microelectronics required to address a big number of pixels.^[^ ^3^ ^]^ For example, thin‐film‐transistor (TFT) active‐matrices have been reported to drive electrochromic displays,^[^ ^4^, ^5^ ^]^ and complementary metal–oxide–semiconductor (CMOS) technology has been used for the integration of high‐throughput electrochemical sensors.^[^ ^6^, ^7^ ^]^ TFT active‐matrix‐driven site‐selective electrochemical reactions have not been well studied so far. However, site‐selective electrochemical processes are essential to prepare dissimilar electroactive materials and structures on a single chip. For instance, electropolymerization on specific pixels could be used to immobilize various enzymes enabling simultaneous sensing of biologically relevant substances (e.g., lactate, glucose, etc.).^[^ ^8^ ^]^ Integrating sensor arrays and electronic muscles in, for example, catheters and implants would take telemedicine to a new level of automation and miniaturization, and the integrated devices can be manipulated to site‐selectively sense and interact with the local environment.^[^ ^9^, ^10^ ^]^ Although great progress has been made toward the integration of heterogeneous materials and structures, it is challenging as frontend fabrication technologies in microelectronics put extremely high demands on materials, fabrication protocols, and processing environments. For example, the integration of heterogeneous conducting polymers on a single chip is of significance for constructing microsystems including sensors, displays, and actuators. Nowadays, photolithography is one of the mainstream technologies for multiple material patterning and integration with high resolution. However, most conducting polymers are not compatible with standard photolithographic processes since their electroactivity would be influenced by the solvents used during the process. Therefore, a complementary approach is required for the on‐chip heterogeneous integration of such materials.

Among a large variety of thin‐film microelectronics, tremendous attention has been paid to TFTs based on amorphous oxide semiconductors and their integrated circuits since the first reports on the remarkable mobility of amorphous indium‐gallium‐zinc oxide (a‐IGZO) TFTs appeared in 2004.^[^ ^11^ ^]^ Since then, a‐IGZO and other amorphous metal oxide semiconductor based TFTs have been widely used in various microelectronic devices such as flat panel displays and image sensors,^[^ ^12^ ^]^ as well as in the field of flexible and wearable electronics.^[^ ^13^, ^14^ ^]^ Integration of single TFTs into large‐area active‐matrix arrays can generate powerful functionalities, and minimize the number of interconnects required to address individual pixels with high spatial resolution and contrast.^[^ ^15^ ^]^ Typical examples are drive circuits in flat panel displays including active‐matrix liquid‐crystal displays (AMLCDs),^[^ ^16^ ^]^ active‐matrix organic light‐emitting‐diode (AMOLED) displays,^[^ ^17^ ^]^ and magnetic sensory systems and matrices.^[^ ^18^ ^]^ From a microelectronics point of view a‐IGZO TFTs are competitive candidates for manufacturing active‐matrix digital devices with good uniformity, high mobility, and low processing temperatures.^[^ ^19^ ^]^ However, a‐IGZO TFTs are, in general, sensitive to moisture and solvents,^[^ ^20^ ^]^ and the performance would quickly degrade when exposed to the electrochemical solutions. Therefore, an efficient device passivation and encapsulation is necessary to operate the a‐IGZO TFTs in electrochemical devices.^[^ ^21^ ^]^

**Figure 1:**
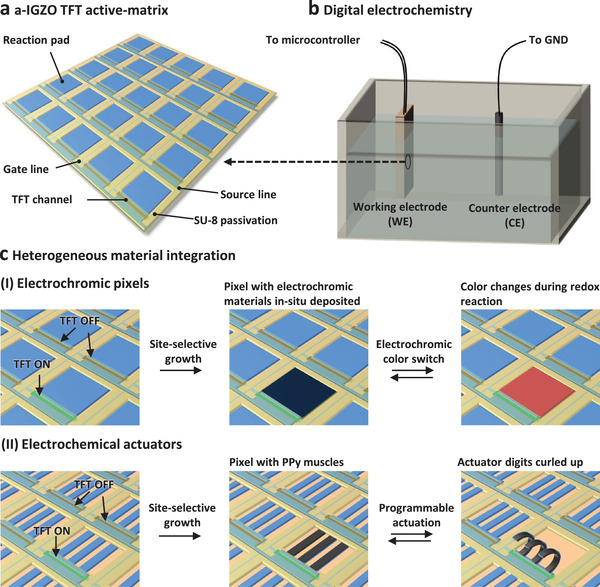
Concept of digital electrochemistry and its application in heterogeneous material integration. a) Schematic illustration of the a‐IGZO TFT active‐matrix for addressing the electrochemical reactions. b) Schematic illustration of the digital electrochemical reaction cell, where the a‐IGZO TFT active‐matrix is used as the working electrode (WE), and the counter electrode (CE) is grounded. c) Site‐selective electrochemical reactions are carried out for in situ functional material deposition and heterogeneous integration. Depending on the functionalities of the locally deposited electroactive materials, I) active‐matrix‐driven electrochromic patterns and II) active‐matrix‐driven PPy actuators are fabricated.

To overcome the obstacle in heterogeneous material integration, here we present a new integration strategy based on digital electrochemistry driven by an on‐chip integrated a‐IGZO TFT active‐matrix. With this approach, heterogeneous materials can be integrated onto the chip after the frontend fabrication of the driving TFT active‐matrix. The digitally controlled and site‐specific electrochemical processes enable integration of dissimilar materials and structures at specific pixels and provide true multi‐functionality (**Figure** **1**). The average mobility of the a‐IGZO TFTs within the matrix is (10.5 ± 0.8) cm^2^ V^−1^ s^−1^ and the on/off ratio reaches (8.4 ± 1.6) × 10^7^, which enables the active switching of pixels between their off and on states for the site‐selective electrochemical reactions. Due to efficient passivation, the a‐IGZO TFT active‐matrix is stable in aqueous and nonaqueous solutions. Site‐selective digital electrochemistry is studied in‐depth by using conducting polymer PPy as a model material. Active‐matrix‐driven electrochromic patterns and electrochemical actuator arrays are fabricated to demonstrate the capabilities of this approach for heterogeneous material integration (Figure 1c). Monochromatic and multicolored electrochromic patterns composed by poly(3,4‐ethylenedioxythiophene) (PEDOT), poly(3‐methylthiophene) (P3MT), and poly(2,5‐dimethoxyaniline) (PDMA) pixels are obtained by site‐selective electropolymerization, and the patterns display different electrochromic color combinations activated with the a‐IGZO TFT active‐matrix (Figure 1c‐I). Active‐matrix‐driven PPy actuator arrays are fabricated by electrodepositing PPy electronic muscles locally, and the actuator pixels can be individually actuated through the active‐matrix (Figure 1c‐II). The digital electrochemistry approach is applicable to a broad range of materials, which would find numerous applications for the heterogeneous integration between electrochemical materials, structures, and microelectronics for near‐future 3D microelectronic systems.^[^ ^22^ ^]^

## 2 Results

### 2.1 Active‐Matrix Fabrication

**Figure 2:**
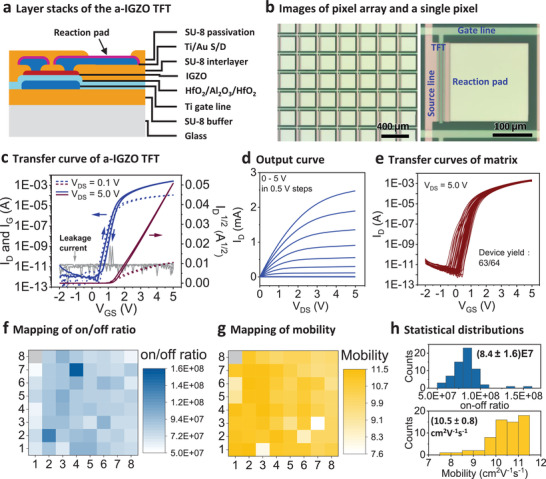
Structure and electrical performance of the a‐IGZO TFT active‐matrix. a) Schematic layer stacks of the a‐IGZO TFTs. b) Optical microscopy images of the pixel array and a single pixel on the active‐matrix. One pixel consists of a control TFT and a reaction pad. The reaction pad is connected to the drain of the TFT. c) Typical transfer characteristics with *V*_GS_ from −2 to 5 V for the a‐IGZO TFTs (*L* = 6.5 µm, *W* = 200 µm). The leakage current is plotted with gray lines. d) Corresponding output characteristics at *V*_GS_ from 0 to 5 V in 0.5 V steps. e) Transfer characteristics of all the 63 working TFTs in the 8 × 8 matrix. f,g) Spatial distributions of on/off ratios and mobilities of the a‐IGZO TFT active‐matrix. The dysfunctional pixel is marked with a gray block. h) Histograms of the on/off ratios and mobilities of the 63 working TFTs showing their statistical distributions. The average on/off ratio is (8.4 ± 1.6) × 10^7^, and the average mobility is (10.5 ± 0.8) cm^2^ V^−1^ s^−1^.

The layer stacks of the a‐IGZO TFT active‐matrix are schematically shown in **Figure** **2**a. Various material deposition methods, such as spin coating, magnetron sputtering, and atomic layer deposition (ALD) were used to deposit the constructing layers. Photolithography together with dry and wet etching methods were employed to pattern individual layers. Details on the step‐by‐step fabrication process are described in the Experimental Section and Figure S1 in the Supporting Information. In the TFT stacks, three SU‐8 layers with different functions were used. The SU‐8 buffer layer was applied to smoothen the glass surface, thereby improving the yield. The 130 nm thick SU‐8 interlayer was patterned to protect the semiconductor channel during dry etching of the Ti/Au source/drain (S/D) layer. The SU‐8 passivation layer covered the TFT channel regions and the addressing lines, leaving the drain Ti/Au pads exposed to electrochemical reactions. The size of the reaction pad is 200 × 200 µm^2^, as shown in Figure 2b. Since the channel regions are isolated by the SU‐8 double layers, the TFTs can be stably operated in aqueous and nonaqueous solutions. A 15 nm thick a‐IGZO film was sputtered and patterned to form the channel layer (Figure S2, Supporting Information). The 200 µm long and 6.5 µm wide channels are defined by the SU‐8 interlayer. A HfO_2_/Al_2_O_3_/HfO_2_ sandwich structure was used as dielectric layer taking advantage of the large bandgap of Al_2_O_3_ and high dielectric constant of HfO_2_.^[^ ^23^ ^]^ Compared to TFTs with just a HfO_2_ or Al_2_O_3_ dielectric layer the sandwich structure can efficiently improve the leakage current and breakdown voltage while keeping small hysteresis in the transfer characteristics (Figure S3, Supporting Information). The dielectric layer (6.2 nm HfO_2_/3.5 nm Al_2_O_3_/6.2 nm HfO_2_) has an equivalent dielectric constant of 15.3 (Figure S4, Supporting Information) and is essential for achieving low‐voltage operation of the a‐IGZO TFTs.^[^ ^24^ ^]^

### 2.2 Electrical Performance

Typical transfer characteristics of the a‐IGZO TFTs are shown in Figure 2c. The source current (*I*_D_) and gate current (*I*_G_, leakage current) are plotted as a function of the gate‐source voltage (*V*_GS_) at source–drain voltages (*V*_DS_) of 0.1 and 5 V. The square root of *I*_D_ is also plotted against *V*_GS_ to extract the threshold voltage (*V*_th_). The on‐current at *V*_GS_ = *V*_DS_ = 5 V reaches 2 mA and the off‐current is as low as several pA leading to an on/off ratio of ≈10^8^. The on‐current at *V*_GS_ = 5 V, *V*_DS_ = 0.1 V is still as high as 0.1 mA. The high on‐current is sufficient for performing electrochemical depositions on the 200 × 200 µm^2^ pads. The leakage current is less than 10 pA, due to the excellent performance of the dielectric layer. There is a small clockwise hysteresis in the subthreshold region, possibly due to a small number of deep‐state traps in the dielectric layer, semiconductor layer or their interface.^[^ ^25^ ^]^ Since the TFTs are used as single digital switches operating at low frequency, this small hysteresis has no influence on our devices. The corresponding output characteristics are shown in Figure 2d. *I*_D_ is plotted with *V*_DS_ by scanning *V*_GS_ from 0 to 5 in 0.5 V steps. The *I*_D_ increases linearly at small *V*_DS_ and saturates beyond the pinch‐off point, exhibiting typical TFT behavior. Active‐matrices with 8 × 8 pixel arrays were fabricated, and the electrical performance of the control a‐IGZO TFT array was characterized. Figure 2e shows the transfer curves of all the 63 working TFTs between −2 and 5 V gate bias. The device yield is around 98%, which is high enough for the proof‐of‐concept demonstrations. Based on the transfer curve test, spatial distributions of TFT performance parameters are plotted in Figure 2f,g and Figure S5 in the Supporting Information, including on/off ratio, mobility, *V*_th,_ and subthreshold swing (SS). Statistical distributions of these parameters are shown in Figure 2h and Figure S5 in the Supporting Information. The narrow statistical distribution of all the performance parameters documents the high uniformity of the TFT array. The high average on/off ratio of (8.4 ± 1.6) × 10^7^ discriminates unambiguously between on and off pixels for the site‐selective electrochemical reactions. The average mobility of (10.5 ± 0.8) cm^2^ V^−1^ s^−1^ is consistent with previously reported values for amorphous IGZO TFTs.^[^ ^26^ ^]^ *V*_th_ of (1.1 ± 0.1) V indicates that the n‐type a‐IGZO TFTs worked in the enhancement mode.^[^ ^13^ ^]^ The low SS of (140.9 ± 8.6) mV dec^−1^ is due to the high dielectric constant of the dielectric layer and the good quality of the dielectric–semiconductor interface.^[^ ^27^ ^]^ Ultralow voltage conditions are highly desirable in some applications, such as wearable or electrochemical devices to avoid parasitic electrochemical reactions, and promote safe and low power operation.^[^ ^28^ ^]^ Due to the ultrathin dielectric layer, our a‐IGZO TFT active‐matrix could work at a low voltage of 1.8 V. The electrical performance of the a‐IGZO TFTs and active‐matrix up to 1.8 V gate bias is shown in Figures S6–S8 in the Supporting Information.

A good stability of the IGZO TFT performance is important for long‐term operation. We therefore carried out an environmental stability test as well as a constant‐voltage‐bias‐stress test. Figure S9 in the Supporting Information shows the transfer curve shift and TFT parameter variations by immersing the IGZO TFTs in deionized (DI) water at 85 °C. After 24 h the TFTs are still working despite local delamination of the SU‐8 passivation layer. During the environmental stability test the mobility decreases constantly from (8.2 ± 0.8) cm^2^ V^−1^ s^−1^ to (6.2 ± 1.4) cm^2^ V^−1^ s^−1^. The on/off ratio drops significantly within the first 3 h and then stabilizes above 1 × 10^6^. *V*_th_ increases by 0.8 V within the first 3 h and then stabilizes at around 2.2 V. The SS value varies within 120 to 140 mV dec^−1^ and does not show any clear trend over time. Figure S10 in the Supporting Information shows the TFT drain current change and transfer curve shift during a constant‐voltage‐bias‐stress test. The TFT is still functioning well after the stress process. The constant‐voltage‐bias stressing results in a positive transfer curve shift and a small decrease of the on‐current. The environmental stability and constant‐voltage‐bias stress‐test results indicate that our IGZO TFTs are sufficiently stable for long‐term operation.

In our laboratory‐scale experiments, 8 × 8 TFT arrays with a fabrication yield of around 98% were used for several proof‐of‐concept demonstrations. In practical applications such as multicolored electrochromic displays^[^ ^5^ ^]^ and high‐throughput electrochemical sensors,^[^ ^7^ ^]^ large‐area arrays with thousands of pixels are required. In these devices, the yield of the TFTs becomes a bottleneck for mass production. In our active‐matrix devices, we employed a simple design by using a single TFT to control each pixel. Since our devices are operated in solution and the pixel pads are all electrically connected by the solution, failure or breakdown of one pixel TFT causes the whole row or column to malfunction. To improve this, a higher level of sophistication and manufacturing precision could be achieved in industrial production lines. Furthermore, cleverly refined pixel and connection designs will help to construct large‐area devices with higher integration density. For example, we can adopt a design that isolates the dysfunctional pixels from the whole system. In this way the device would still work well even though a small number of dysfunctional pixels are lost.

### 2.3 Digital Electrochemistry

**Figure 3:**
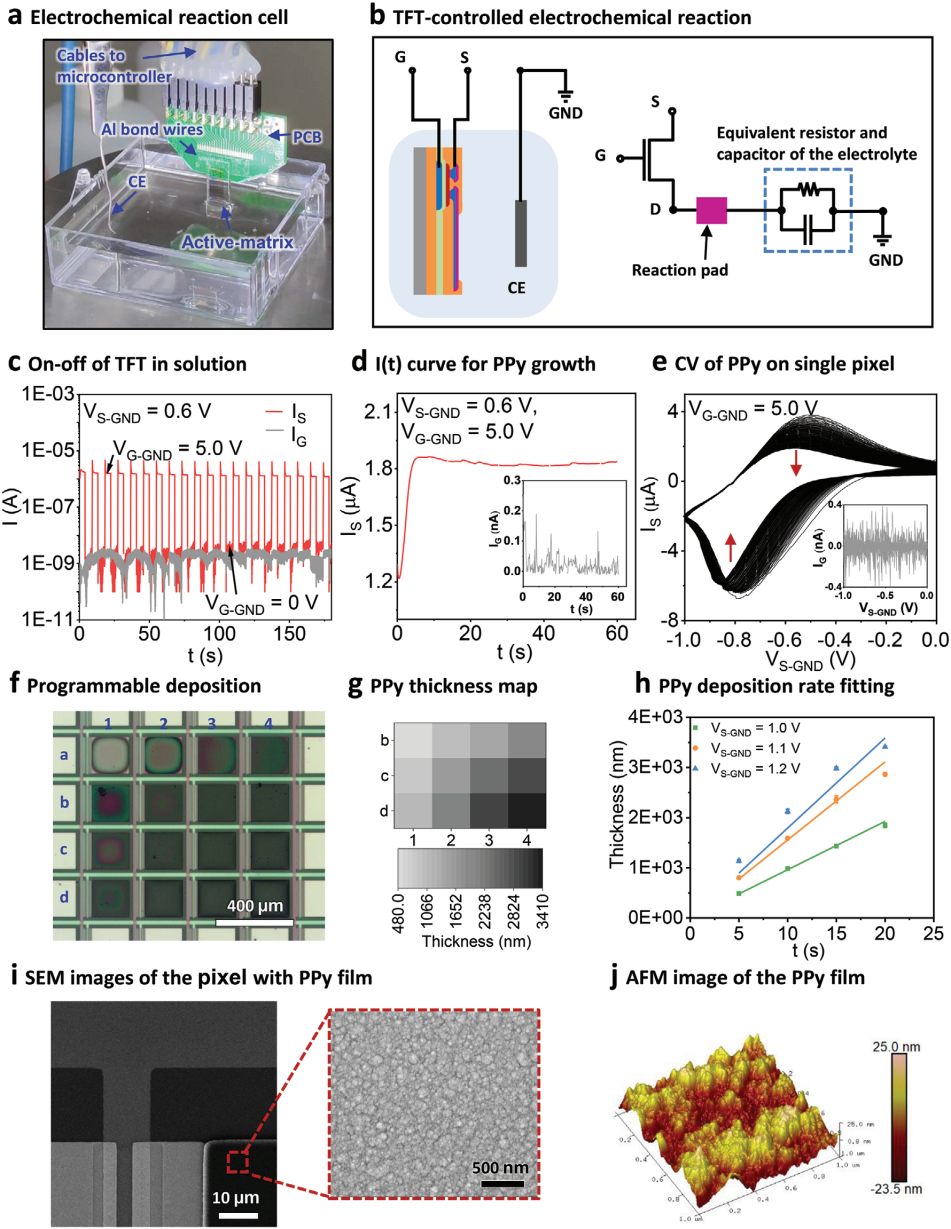
Digital electrochemistry of PPy on the a‐IGZO TFT active‐matrix. a) Photograph of a two‐electrode electrochemical reaction cell. b) Schematic and circuit diagrams for the TFT‐controlled electrochemical reaction. c) *I*(*t*) curve for the on‐off operation of the a‐IGZO TFT in the PPy monomer solution. The *V*_S‐GND_ is kept as 0.6 V versus Ag/AgCl and the control TFT is turned on and off by switching the *V*_G‐GND_ between 5 and 0 V. d) Typical *I*(*t*) curve for the PPy deposition on a single pixel. *V*_G‐GND_ = 5 V and *V*_S‐GND_ = 0.6 V versus Ag/AgCl. The inset shows the leakage current *I*_G_ plotted versus deposition time. e) CV of the deposited PPy on a single pixel. The *V*_G‐GND_ is kept as 5 V. The inset shows the leakage current *I*_G_ during the voltage scan. f) Optical microscopy image showing a 4 × 4 array with PPy programmatically deposited. From row a to row d, the *V*_S‐GND_ is 0.9, 1.0, 1.1, and 1.2 V versus Au, respectively, and from column 1 to 4 the deposition time is 5, 10, 15, and 20 s, respectively. For all the depositions the *V*_G‐GND_ is fixed as 5 V. g) Thickness mapping of the deposited PPy corresponding to (f). h) Plot and linear fitting of the PPy thickness versus deposition time at different *V*_S‐GND_. i,j) Typical SEM images (SE contrast) and AFM image of the as‐deposited PPy film on the pixel.

PPy was used as a model material to demonstrate the concept of digital electrochemistry, since it is a well‐known conducting polymer widely used in electrochemical sensors and actuators.^[^ ^29^, ^30^, ^31^ ^]^ **Figure** **3**a shows a two‐electrode electrochemical reaction cell, where the a‐IGZO TFT active‐matrix is used as the working electrode (WE), and Ag/AgCl is used as a counter electrode (CE). We used pyrrole monomer aqueous solution as the electrolyte containing 0.1 m pyrrole and 0.1 m sodium dodecylbenzenesulfonate (NaDBS).^[^ ^29^ ^]^ A schematic and a circuit diagram of the TFT‐controlled electrochemical reaction are shown in Figure 3b. The reaction pads are connected to the drain sides of the TFTs and the CE is grounded. When the TFT is switched on by applying a gate bias, current flows from the source line to the CE through the TFT channel and the electrolyte. In its “On” state, the TFT has a channel resistance orders of magnitude smaller than the equivalent resistance of the electrolyte. Therefore, the main voltage drop is through the electrolyte, and a potential is built between the reaction pad and the CE. Electrochemical reactions take place once the potential exceeds a threshold value. The pyrrole monomers are electropolymerized when the potential is higher than 0.55 V versus Ag/AgCl.^[^ ^30^ ^]^ When the TFT is switched off the electrochemical reaction stops immediately. More details on the concept of TFT‐controlled digital electrochemistry and the test setup are discussed in Note S3 in the Supporting Information.

To evaluate the switch function of the control a‐IGZO TFT, on‐off operation was performed in the pyrrole monomer solution as shown in Figure 3c. The source voltage *V*_S‐GND_ was kept as 0.6 V versus Ag/AgCl by considering a small voltage drop through the TFT channel and the connections. The control TFT was switched between on and off by alternatively setting the gate voltage *V*_G‐GND_ between 5 and 0 V (vs grounded Ag/AgCl CE) every 4 s. A substantial deposition current *I*_S_ in the microampere range is observed at *V*_G‐GND_ = 5 V, and *I*_S_ drops to the nanoampere range once *V*_G‐GND_ = 0. During the operation, the leakage current *I*_G_ is as low as several nanoampere. This result shows that the PPy deposition could be effectively controlled through the switching of the control TFT. Note that there is a small spike on the *I*_S_(*t*) curve immediately after the TFT is switched on. This is due to the fast charging of the electrolyte equivalent capacitance.^[^ ^29^ ^]^ Here, potentiostatic deposition of PPy is adopted as a typical electrochemical reaction. Figure 3d shows the *I*_S_(*t*) curve for the deposition of PPy on a single pixel. The deposition current increases dramatically during the first several seconds and stabilized at around 1.82 µA, giving a current density of 4.55 mA cm^−2^. Cyclic voltammetry (CV) was performed on the same pixel to check the quality of the deposited PPy, as shown in Figure 3e. A pair of reduction and oxidation peaks located at around −0.8 and −0.5 V versus Ag/AgCl can be clearly seen from the 100 cycles of the scan, indicating that the deposited PPy is electroactive during the redox reaction. The reduction and oxidation peak values decrease slightly with the increase of the scan cycles. This cycling instability is probably due to the detachment of PPy from the surface of the Ti/Au reaction pad. During the CV cycling the PPy film undergoes significant volume change and large repeated stress is induced at the PPy–Au electrode interface which leads to delamination of the PPy film from the electrode.^[^ ^32^ ^]^ PPy actuators take advantage of this very expansion and contraction during the redox process and translate it to a bending deformation with high curvature. However, the volume change is detrimental to other electrochemical devices such as electrochromic displays, supercapacitors, and batteries. As reported by Liu et al.,^[^ ^32b^ ^]^ various processes which result in increased electrode surface roughness can enhance the adhesion of PPy to the underlying electrodes and improve the cycling stability. The leakage currents during the PPy deposition and CV scan were recorded as shown in the insets in Figure 3d,e. The ultralow leakage current reveals the high stability of the TFTs in the solution.

To demonstrate the high controllability of the digital electrochemistry, programmable deposition of PPy addressed by the a‐IGZO TFT active‐matrix was carried out with the assistance of a shift register and a digital‐to‐analog‐converter (DAC) voltage source which were driven by a microcontroller for pixel selection (Figure S11, Supporting Information). PPy was deposited on a 4 × 4 array in a pixel‐wise manner (Figure 3f; Movie S1, Supporting Information). Deposition parameters, including the source bias and the deposition time were programmatically controlled to obtain a gray scale pattern of PPy films. The thickness map of the PPy array is shown in Figure 3g. The PPy thickness varies over a range from 480 nm (Pixel 1b, *V*_S‐GND_ = 1.0 V, *t* = 5 s) to 3.4 µm (Pixel 4d, *V*_S‐GND_ = 1.2 V, *t* = 20 s) (Note pixels in row a are not shown due to the inhomogeneity of the film thickness). Figure 3h shows the plot of PPy thickness versus deposition time at different source biases. The thickness increases linearly with deposition time, with a rate of 94.3 nm s^−1^ at 1.0 V, 150.2 nm s^−1^ at 1.1 V, and 204.8 nm s^−1^ at 1.2 V (the source bias was vs Au in this experiment). The deposited PPy films show typical agglomerated granular‐like morphology,^[^ ^33^ ^]^ as shown by the scanning electron microscopy (SEM) and atomic force microscopy (AFM) images in Figure 3i,j and Figure S12 and Figure S13 in the Supporting Information. The surface roughness increases slightly from 6.2 nm and stabilizes at 7.4 nm with increasing deposition time (Figure S13, Supporting Information). Besides the programmable deposition of PPy, the active‐matrix could also be used for electrochemical deposition of many other materials. Movie S2 in the Supporting Information shows the electroplating of Ni patterns on the device from a commercial Ni electroplating solution.

### 2.4 Electrochromic Patterns

**Figure 4:**
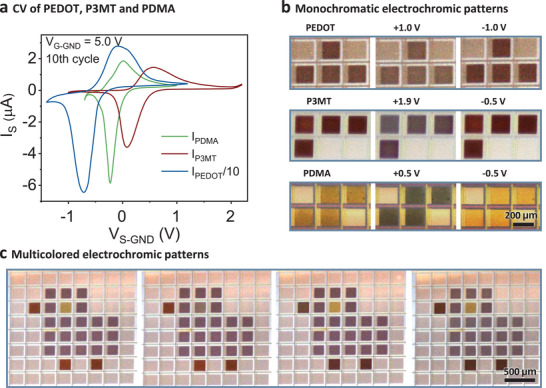
Site‐selective deposition and color switch of multicolored electrochromic patterns. a) CV of PEDOT, P3MT, and PDMA on individual active‐matrix pixels. The *V*_G‐GND_ is kept as 5 V during the source bias scan. b) Optical microscopy images of PEDOT, P3MT, and PDMA patterns in the as‐deposited, oxidation and reduction states, respectively. For the oxidation and reduction states, the applied *V*_S‐GND_ are marked on top of the microscopy images. c) PEDOT, P3MT, and PDMA are sequentially deposited on the same active‐matrix and the pixels within the pattern can be changed between different electrochromic colors switched by the a‐IGZO TFT active‐matrix. The PDMA eye displays color changes between yellow and green, the P3MT beak and legs show color changes between red and dark blue, and the PEDOT body shows colors between dark blue and light blue.

Conducting polymers, such as polythiophene (PTh) and polyaniline (PANI) and their derivatives are a class of electrochromic materials which change their colors during redox reactions, and therefore are widely used as active components in electrochromic devices.^[^ ^34^ ^]^ Electrochromic conducting polymers including PEDOT, P3MT, and PDMA^[^ ^35^ ^]^ are site‐selectively polymerized on the a‐IGZO TFT active‐matrix to demonstrate the versatility of the digital electrochemistry approach in the integration of multicolored electrochromic devices. PEDOT, P3MT, and PDMA were first deposited separately on individual pixels, and their electrochemical properties were evaluated by CV, as shown in **Figure** **4**a and Figure S14 in the Supporting Information. Pairs of reduction and oxidation peaks were observed for each material, with locations at −0.7 and −0.08 V versus Ag/AgCl for PEDOT, 0.08 and 0.55 V versus Ag/AgCl for P3MT, and −0.23 and 0.01 V versus Ag/AgCl for PDMA. PEDOT and P3MT were deposited and characterized in a propylene carbonate (PC) solution, whereas PDMA was deposited and characterized in a water‐based solution because PDMA would be dissolved in the PC based solution. The leakage currents in all cases are no higher than several nA which did not increase during 100 scan cycles, indicating that the devices are highly stable in aqueous and nonaqueous solutions.

Digitally controlled electropolymerization of the electrochromic materials on multiple pixels is driven by the a‐IGZO TFT active‐matrix, similar to the programmable deposition of PPy patterns. PEDOT, P3MT, and PDMA monochromatic patterns were deposited in their respective monomer solutions. After deposition, a given source bias was applied to specific pixels for reversible oxidation or reduction of the materials on top of the pixels to observe the electrochromic color change. Under different redox states, the electrochromic patterns display different colors and contrasts, as shown in Figure 4b and Movie S3 in the Supporting Information. The color change could take place on a single pixel or simultaneously on multiple pixels. Heterogeneous multicolored electrochromic patterns could also be obtained by sequential deposition (Movie S4, Supporting Information). A colorful bird pattern consisting of a PEDOT body, P3MT beak and legs, and PDMA eye is displayed in Figure 4c. The pixels in the pattern show various color combinations digitally modulated by the a‐IGZO TFT active‐matrix (Movie S5, Supporting Information). The PDMA eye displays color changes between yellow and green, the P3MT beak and legs show color changes between red and dark blue, and the PEDOT body shows color changes between dark blue and light blue, depending on the bias applied to the corresponding pixels.

### 2.5 Active‐Matrix PPy Actuators

**Figure 5:**
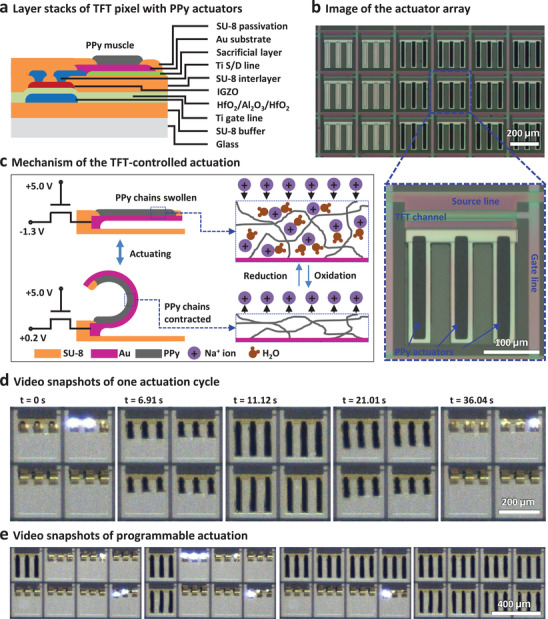
Fabrication and actuation of the active‐matrix PPy actuator arrays. a) Schematic layout of the a‐IGZO TFT pixel with PPy actuators. b) Optical microscopy images of the PPy actuator array and a single pixel. The PPy actuator digits are connected to the drain of the TFT. c) Schematic illustration of the actuation mechanism. With the control TFT switched on, the PPy is in different redox states depending on the applied source bias, which leads to the absorbing and expelling of Na^+^ ions and water molecules. PPy muscles have a volume change and the digits are actuated. d) A series of video snapshots showing a 2 × 2 array with PPy actuators in one actuation cycle. e) Video snapshots of a 4 × 2 actuator array in different states during the programmable actuation.

PPy has been widely used as artificial muscles in bioinspired actuators due to its good chemical stability and large strain which can be induced by low voltages (<1 V).^[^ ^36^ ^]^ Integrating the PPy electronic muscles into next‐generation microelectronic catheters would allow the device to be manipulated in complex environment.^[^ ^10^ ^]^ Recently, our group has reported a PPy‐based intelligent actuator system with position and strain feedback control.^[^ ^31^ ^]^ Here, a‐IGZO TFT active‐matrix‐driven PPy actuator arrays were fabricated to demonstrate the capability of the digital electrochemistry approach in electrochemical actuator integration. To construct actuator pixels, the metal reaction pads were replaced by SU‐8 passivated Au stripes. An organometallic sacrificial layer (SL) was patterned in advance under the Au/SU‐8 bilayer,^[^ ^37^ ^]^ as schematically illustrated in **Figure** **5**a. Each Au stripe has a dimension of 200 × 40 µm^2^, with a 25 × 40 µm^2^ area overlapped with the TFT drain side. A 400 nm thick SU‐8 film was patterned on top of the Au stripes, which served as passivation layer and mechanical support for the actuator digits, leaving a 175 × 20 µm^2^ via hole for PPy muscle deposition. Figure 5b shows the PPy actuator array and a single pixel before the selective removal of the SL. The thickness of the PPy muscles was controlled at around 2.8 µm. After etching of the SL, freestanding actuator digits with one end anchored to the drain of the control TFT were obtained. Further details on the step‐by‐step fabrication and structural characterization of the PPy actuator arrays are shown in Figure S15 in the Supporting Information.

Programmable actuation of the PPy actuator array was carried out in 0.1 m NaDBS solution. A schematic illustration of the TFT‐controlled actuation mechanism is given in Figure 5c. To actuate a specific pixel, a fixed gate bias of 5 V was applied to switch on the control TFT, and a variable source bias was applied to determine the redox state of the PPy muscles. When the source bias is −1.3 V versus Ag/AgCl, PPy is reduced and swells due to the influx of hydrated Na^+^ ions. The swelling of the conducting polymer reshapes the actuator digits into a flat state. When the source bias is increased to 0.2 V versus Ag/AgCl, PPy is oxidized and shrinks by repelling Na^+^ ions and water out of the film. In this case, the actuator digits are bent up by the contracting force of the PPy muscles.^[^ ^38^ ^]^ Therefore, a periodic flattening and bending motion of the actuator digits could be observed due to the reversible volume change of the PPy muscles upon scanning the source bias. Figure 5d shows a series of video snapshots for a 2 × 2 actuator array over one actuation cycle (Movie S6, Supporting Information). The source bias is scanned from 0.2 to −1.3 V versus Ag/AgCl with a scan rate of 100 mV s^−1^. The actuator digits are initially in the curled state, then flatten out after around 11 s, and bend up again to the initial state. The duration of one actuation cycle is 30 s, depending on the source bias scan rate. The periodic actuation could be repeated many times (Movie S7, Supporting Information). Programmable actuation is achieved by addressing individual actuator pixels through the a‐IGZO TFT active‐matrix. Figure 5e shows the video snapshots of a 4 × 2 PPy actuator array in different states during the programmable actuation (Movie S8, Supporting Information). The actuators can be manipulated in a pixel‐wise way or simultaneously actuated, depending on the addressing bias. So far, all the activated actuators have a synchronized motion. More sophisticated actuation of the array is possible by programming the input bias signals.

## 3 Conclusions

We have introduced a new approach for on‐chip heterogeneous material and structure integration by carrying out site‐selective electrochemistry triggered and addressed by a‐IGZO TFT active‐matrices. The digital electrochemistry approach is used to generate electroactive materials locally and introduce versatile functionalities to the electrochemical device pixels. Active‐matrix‐driven multicolored electrochromic patterns and active‐matrix PPy actuator arrays have been fabricated. This approach is applicable to a broad range of materials and has tremendous potential for the fabrication and integration of electrochemical devices for parallel synthesis, multicolored electrochromic displaying, electrochemical actuation, and high‐throughput analysis, as well as microelectronic catheters, implants, and electronic skins to name a few. The demonstration of the digital electrochemistry concept has brought together microelectronic engineers and electrochemists, and future joint efforts between these two communities are expected to push forward and explore a broad range of new practical applications based on large‐scale integrated heterogeneous device architectures.

## 4 Experimental Section

### Fabrication of the a‐IGZO TFT Active‐Matrix

Glasses with dimensions of 50 × 50 mm^2^ and a thickness of 1 mm were used as handing substrates. The glasses were washed in a Miele professional washer (Miele & Cie. KG, Gütersloh, Germany) and treated with oxygen plasma in the GIGAbatch 310m (PVA Metrology & Plasma Solutions GmbH, Wettenberg, Germany). SU‐8 2000.5 (300 nm) was spin‐coated and photopatterned as a buffer layer. Ti (100 nm) gate layer was DC magnetron sputtered (HZM‐4P, IvA, Dresden, Germany) and wet etched in 0.1 m NaF and 0.1 m (NH_4_)_2_S_2_O_8_ solution. Dielectric layer of 6.2 nm HfO_2_/3.5 nm Al_2_O_3_/6.2 nm HfO_2_ was deposited by plasma‐enhanced atomic layer deposition (PEALD, FlexAL, Oxford Instruments PLC, Abingdon, UK), and dry‐etched with CF_4_ and Ar chemistry by reactive ion etching (RIE, Plasma Lab 100; Oxford Instruments PLC, Abingdon, UK). a‐IGZO semiconductor layer (15 nm) was RF magnetron sputtered by a house‐made sputtering machine and wet‐etched in a 4 wt% oxalic acid solution. SU‐8 2000.5 interlayer was spin‐coated, patterned, and trimmed down to 130 nm with oxygen plasma. Ti/Au 25 nm/30 nm source–drain layer was RF magnetron sputtered and dry‐etched with the CF_4_ and Ar chemistry. Finally, the device was passivated by a layer of SU‐8 2000.5 (300 nm), leaving the deposition and contact pads exposed (details on the device fabrication are provided in Note S1, Supporting Information).

### Electrical and Structural Characterization

The transfer and output characteristics of the a‐IGZO TFTs were measured on an automatic probe station (Summit 12000; Cascade Microtech Inc., Beaverton, OR, USA) connected to a Precision Source/Measure Unit (B2902A, Agilent Technologies Inc., Santa Clara, CA, USA). A Precision LCR Meter (E4980A, Agilent Technologies Inc., Santa Clara, CA, USA) was utilized for the capacitance measurements of the Ti/HfO_2_/Al_2_O_3_/HfO_2_/Ti/Au metal–insulator–metal (MIM) structures. SEM characterization was performed using DSM982 GEMINI and NVsion40 (both Carl Zeiss Microscopy GmbH, Jena, Germany). The focused ion beam (FIB) cuts were made in the NVision40 using Ga^+^ ions of 30 kV. AFM characterization was performed on an ICON AFM (Bruker, Billerica, MA, USA). Profile and thickness of the stack layers were obtained on a DekTak profilometer (Bruker, Billerica, MA, USA).

### Digital Electrochemical Deposition of Conducting Polymers

The electrochemical deposition of conducting polymers, including PPy, PEDOT, P3MT, and PDMA was carried out in their respective monomer solutions. To perform site‐selective deposition, TFTs on specific pixels were switched on with a fixed gate bias of 5 V; meanwhile a deposition voltage was applied to the corresponding source lines for a certain time (details are provided in Notes S3 and S4 in the Supporting Information). The deposition process was in situ recorded by a Leica camera (Leica Microsystems GmbH, Wetzlar, Germany).

### Electrochromic Color Change

Digitally controllable electrochromic color change was performed through a microcontroller in a two‐electrode system, where the a‐IGZO TFT active‐matrix with electrochromic material patterns was connected to the WE, and Ag/AgCl was used as the CE. To change the color on specific pixels, 5 V bias was applied to the corresponding gate lines and a given redox potential was applied to the corresponding source lines. The color change process was recorded by a Leica camera (details are provided in Note S4 in the Supporting Information).

### Fabrication of PPy Actuator Arrays

The PPy actuator arrays were fabricated based on the a‐IGZO TFT active‐matrix. After the a‐IGZO TFT active‐matrix was fabricated, a lanthanum‐acrylic acid‐based organometallic photopatternable complex (250 nm) was patterned on top to form a SL. Then an Au layer (50 nm) was DC magnetron sputtered and wet etched. Subsequently, a SU‐8 2000.5 passivation layer (400 nm) was spin‐coated and photopatterned. Finally, PPy muscles were electrochemically deposited and the SL was etched in 1.5 wt% HCl solution to obtain the PPy actuator arrays (details are provided in Note S5 in the Supporting Information).

### Actuation of the PPy Actuator Arrays

Actuation of the PPy actuator arrays was carried out in 0.1 m NaDBS solution. The specific pixels for actuating were digitally addressed by activating the control TFTs through the microcontroller. The source bias was scanned from −1.3 to 0.2 V with a rate of 100 mV s^−1^. During the scan the motion of the actuators was recorded by a Leica camera.

## Conflict of Interest

The authors declare no conflict of interest.

## Supporting information

Supporting Information

Supplemental Movie 1

Supplemental Movie 2

Supplemental Movie 3

Supplemental Movie 4

Supplemental Movie 5

Supplemental Movie 6

Supplemental Movie 7

Supplemental Movie 8

## Data Availability

The data that support the findings of this study are available from the corresponding author upon reasonable request.
