# Supplementary material for: Digital Electrochemistry for On‐Chip Heterogeneous Material Integration
Source: Adv Mater. 2021 May 24;33(26):2101272. doi: 10.1002/adma.202101272 (PMC11469128; doi:10.1002/adma.202101272)
Supplement: Supplementary file 1 — Supporting Information [file ADMA-33-2101272-s008.pdf]

© 2021 Wiley-VCH GmbH

ADVANCED MATERIALS

Supporting Information

for *Adv. Mater.,* DOI: 10.1002/adma.202101272

Digital Electrochemistry for On-Chip Heterogeneous Material Integration

Bin Bao, Boris Rivkin, Farzin Akbar, Dmitriy D. Karnaushenko, Vineeth Kumar Bandari, Laura Teuerle, Christian Becker, Stefan Baunack, Daniil Karnaushenko,* and Oliver G. Schmidt**

Supporting Information

Digital electrochemistry for on-chip heterogeneous material integration

Bin Bao, Boris Rivkin, Farzin Akbar, Dmitriy D. Karnaushenko, Vineeth Kumar Bandari, Laura Teuerle, Christian Becker, Stefan Baunack, Daniil Karnaushenko* & Oliver G. Schmidt**

The PDF file includes:
Supplementary Figures 1-15;
Supplementary Notes 1-5;
Supplementary References 1-8.

Other Supplementary Materials for this manuscript include the following:

Supplementary Movie 1. Digital electrochemical deposition of PPy.
Supplementary Movie 2. Digital electroplating of Ni metal patterns.
Supplementary Movie 3. Electrochromic color change of conducting polymer patterns.
Supplementary Movie 4. Deposition of multicolored electrochromic patterns.
Supplementary Movie 5. Electrochromic color change of multicolored patterns.
Supplementary Movie 6. One cycle actuation of a 2 × 2 actuator array.
Supplementary Movie 7. Repeated actuation of a single actuator pixel.
Supplementary Movie 8. Programmable actuation of a 4 × 2 actuator array.

Supplementary Notes

Supplementary Note 1 Fabrication of the a-IGZO TFT active-matrix

(1) Treatment of the substrates

The fabrication of the a-IGZO active-matrix began with the treatment of glass substrates. Glasses with dimensions of 50 × 50 mm^2 and a thickness of 1 mm (D263T eco glass, SCHOTT AG, Mainz, Germany) were used as handing substrates. The glasses were washed in a professional washer to remove any contaminants and then treated with oxygen plasma to enhance the adhesion of the SU-8 film. SU-8 2000.5 (300 nm, Micro Chem, MA, USA) was spin-coated with a rate of 6500 rpm and photopatterned as a buffer layer to smoothen the glass substrates. The SU-8 buffer layer was completely cross-linked by hard baking at 220°C for 10 min.

(2) Fabrication of the a-IGZO TFT active-matrix for electrochemical reactions

The step-by-step fabrication process of the a-IGZO TFT active-matrix is illustrated in Supplementary Figure S1a. On top of the SU-8 buffer layer, a Ti (100 nm) layer was DC magnetron sputtered and used as back-gate electrodes (gate lines), which were defined by photolithography and subsequently wet-etched in 0.1 M NaF (VWR GmbH, Darmstadt, Germany) and 0.1 M $(NH_4)_2S_2O_8$ (VWR GmbH, Darmstadt, Germany) solution. The gate dielectric layer consisted of a sandwich structure of 6.2 nm HfO_2/3.5 nm Al_2O_3/6.2 nm HfO_2 using plasma-enhanced atomic layer deposition (PEALD) in one batch at 220°C. To access the gate contact pads through the dielectric layer, via holes were dry-etched with CF_4 and Ar chemistry in a reactive ion etching (RIE) machine. The etching depth was carefully controlled to totally etch through the oxide layer but with the underneath Ti layer not etched. a-IGZO semiconductor layer with a thickness of 15 nm was RF magnetron sputtered in a housemade sputtering machine in an Ar and O_2 mixture atmosphere (Supplementary Figure S2).[1] The Ar

flow rate was 30 sccm and O_2 flow rate was 0.5 sccm. The base pressure was around 1×10^{-7} mbar and the deposition pressure was 5×10^{-3} mbar. The sputtering power was 150 W and the sputtering time was 5 min. A three-inch ceramic InGaZnO target (Sindlhauser Materials, Kempten, Germany) with a stoichiometric composition of In:Ga:Zn:O = 1:1:1:4 was used for the sputtering. The a-IGZO film was patterned by wet etching in a 4 wt% oxalic acid (Merck KGaA, Darmstadt, Germany) solution. Subsequently, a second layer of SU-8 2000.5 was spin-coated and patterned as an interlayer (etching stopper layer). The SU-8 interlayer was trimmed down to around 130 nm with oxygen plasma and then hard baked at 220°C for 10 min. 25 nm Ti/30 nm Au source drain layer was RF magnetron sputtered and dry-etched with the CF_4/Ar chemistry. Finally, another layer of SU-8 2000.5 (300 nm) was spin-coated and patterned as passivation layer.[2] The finished sample was hard baked at 220°C for 10 min to totally cross-link the SU-8 passivation layer, as well as enhance the source-drain conductivity. For patterning of all the layers, standard UV photolithography was performed on a mask aligner (MA6, Karl Suss KG-Gmhb & Co, Munich-Garching, Germany). The masks were produced by direct writing on a maskless aligner (MLA 100, Heidelberg Instruments Mikrotechnik GmbH, Heidelberg, Germany). Optical and SEM images of the a-IGZO TFT active-matrix are shown in Supplementary Figure S1b-d.

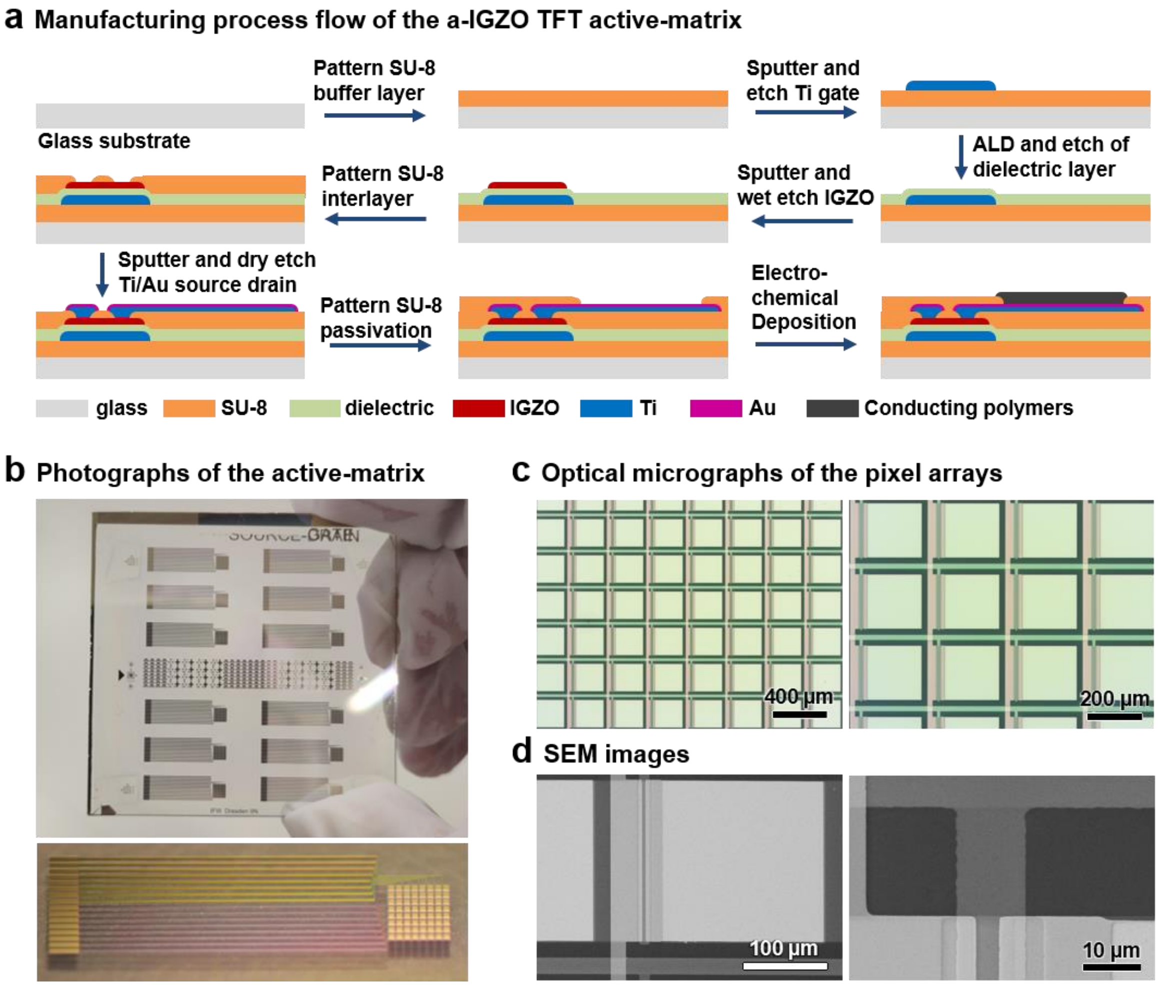

Figure S1. Fabrication and structure of the a-IGZO TFT active-matrix. a) Manufacturing process flow of the a-IGZO TFT active-matrix. b) Up panel: photograph of a 50 × 50 mm^2 sample with 12 devices and various test structures. Bottom panel: photograph of a single device. c) Optical micrographs of the pixel arrays within the device. d) Left: SEM image (SE contrast) of a single pixel containing a control TFT and a reaction pad. Right: SEM image (SE contrast) of the TFT channel region.

(3) AFM characterization of the a-IGZO semiconductor film

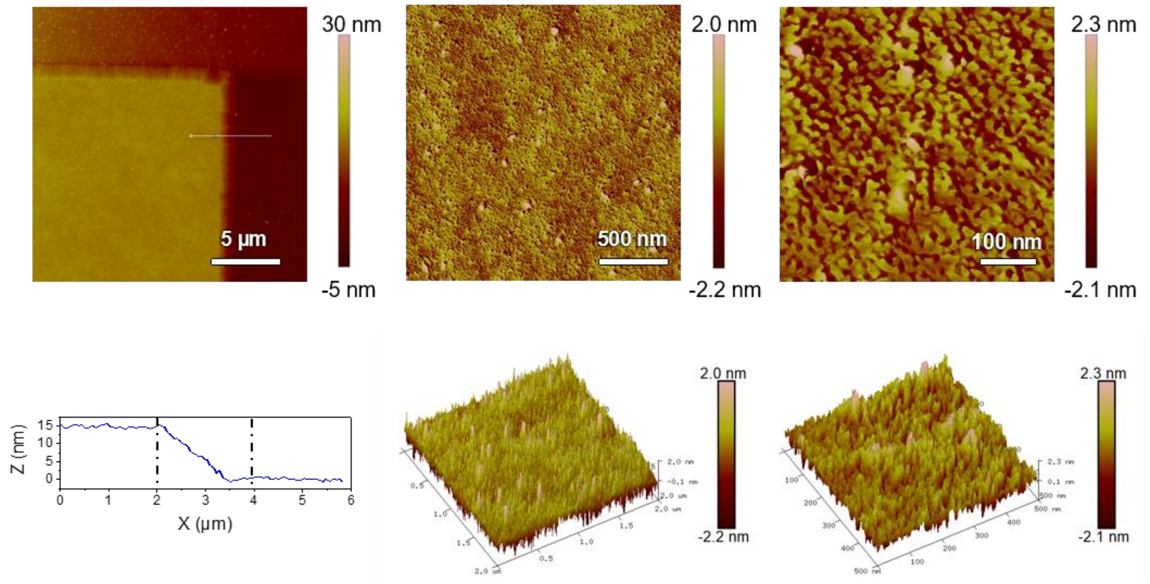

Figure S2. AFM characterization of the sputtered a-IGZO film. The thickness of the a-IGZO film is 15 nm, and the surface roughness is around 0.63 nm. The substrate for the a-IGZO film is with glass/SU-8/HfO_2/Al_2O_3/HfO_2 stack.

Supplementary Note 2 Electrical characterization of the a-IGZO TFTs and active-matrix

(1) Atomic layer deposition and electrical performance of the HfO_2/Al_2O_3/HfO_2 dielectric layer

Atomic layer deposition of the dielectric layer

HfO_2/Al_2O_3/HfO_2 sandwich structure was used as the dielectric layer taking advantage of the large bandgap of Al_2O_3 and the high dielectric constant of HfO_2.[3] The HfO_2 and Al_2O_3 layers were alternatively deposited through PEALD in one batch by using trimethylaluminum (TMA) and tetrakisdimethylamidohafnium IV (TDMAH) as precursors respectively. Before deposition, the temperature of the TDMAH tank was raised to 75°C and stabilized for 1h. The substrate temperature was maintained at 220°C. Ar was used as the carrying and purging gas for the precursors, and the flow rate was fixed at 160 sccm. For one cycle deposition of HfO_2, the TDMAH dose time was 150 ms, and the purge time was 10 s. During the dose process, the chamber pressure was set as 0.1 mbar. The dose process was followed by an O_2 injection of 3 s, with a flow rate of 60 sccm. Then, an O_2 plasma was ignited for 4 s with a power of 400 W. The chamber pressure was kept at 0.02 mbar during the plasma process. Then a post plasma

purge of 10 s was carried out with Ar gas. This cycle was repeated until the HfO_2 thickness reached certain value. For the PEALD of Al_2O_3, the only difference from HfO_2 PEALD process was that the TMA dose time was set as 40 ms. For determining the deposition rate, the thickness of the deposited film was in-situ monitored by an ellipsometer every 10 cycles. In our experiments, the HfO_2 deposition rate was 1.23 Å/cycle, and the Al_2O_3 deposition rate was 1.16 Å/cycle. A three-segment deposition of 50 cycles of HfO_2, 30 cycles of Al_2O_3 and 50 cycles of HfO_2 resulted in a dielectric film consisting of 6.2 nm HfO_2/3.5 nm Al_2O_3/6.2 nm HfO_2.

For comparison, TFTs with just 200 cycles of HfO_2 or Al_2O_3 as the dielectric layers are also fabricated. From the transfer characteristics in Supplementary Figure S3 we can see that TFTs with just HfO_2 as the dielectric layers have small hysteresis in transfer curves but big gate leakage current at elevated (over 1.8 V) gate voltages (Supplementary Figure S3a). The small hysteresis indicates that the defect density in the HfO_2 dielectric layer and the HfO_2-IGZO interface is small. The large leakage current is probably due to the crystallization of HfO_2 and pinhole formation during the ALD process. The TFTs with just Al_2O_3 as the dielectric layer show large hysteresis in the transfer curves (Supplementary Figure S3b), possibly due to the defects within our ALD Al_2O_3 layer or at the Al_2O_3-IGZO interface. This hysteresis cannot be removed even with post annealing at 220°C for one hour (critical temperature for our polymeric layers). Therefore, we added a thin layer of Al_2O_3 in the HfO_2 layer to prohibit the crystallization of HfO_2, thus improving the leakage current and breakdown voltage while keeping small hysteresis in the transfer characteristics.

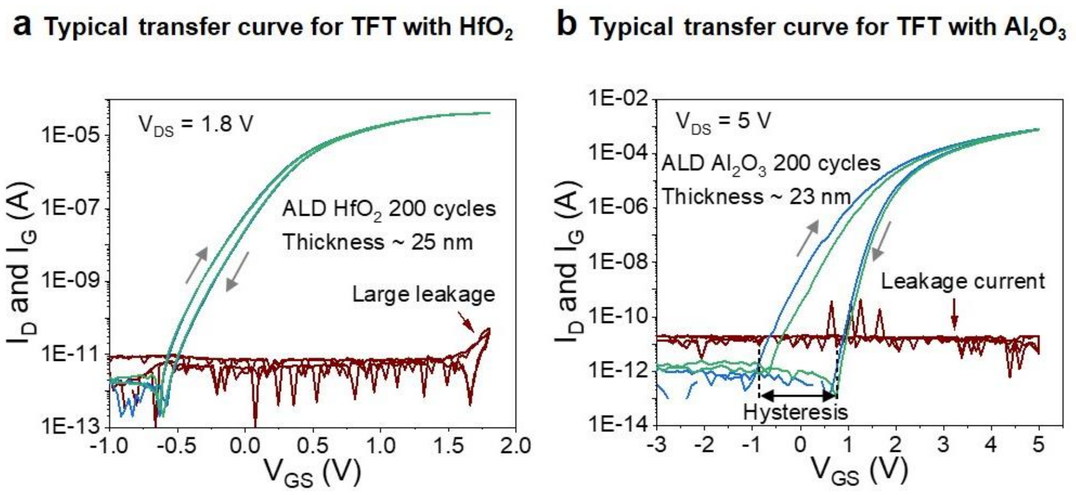

Figure S3. Typical transfer characteristics of TFTs with just HfO_2 or Al_2O_3 as gate dielectric layer. a) Typical transfer curve of TFTs with 25-nm-thick HfO_2 dielectric layer. The hysteresis is small but the leakage current increases with the gate voltage. b) Typical transfer curve of TFTs with 23-nm-thick Al_2O_3 dielectric layer. The leakage current is small but the hysteresis is as large as 2 V.

Electrical performance of the dielectric layer

Electrical characterization of the dielectric layer was carried out on Ti/HfO_2/Al_2O_3/HfO_2/Ti/Au MIM structures[4] with an area of 200 × 200 μm^2. The leakage current density was recorded vs. electric field and voltage as shown in Supplementary Figure S4b. The breakdown field was large than 6 MV/cm and the breakdown voltage was as high as 10 V for the 15.9 nm dielectric trilayer. This was due to the large bandgap of the Al_2O_3 layer. The areal capacitance vs. frequency at different bias and areal capacitance vs. voltage at 1 kHz were plotted in Supplementary Figure S4c and d. The average areal capacitance was taken from -1 V to 1 V at 1 kHz, and calculated as (8.57 ± 0.02) × 10^{-7} F/cm^2. Based on the areal capacitance and thickness of the dielectric layer, the calculated dielectric constant was as high as 15.3. The theoretical dielectric constant of the 6.2 nm HfO_2/3.5 nm Al_2O_3/6.2 nm HfO_2 in serial stack varied from 13.2 to 18.0, by taking the dielectric constant of HfO_2 as 20 to 25, and the dielectric constant of Al_2O_3 as 6-9.[5] Our experimental result was within the theoretical range. The high dielectric constant of the dielectric layer was the key for the low-voltage TFT operation.

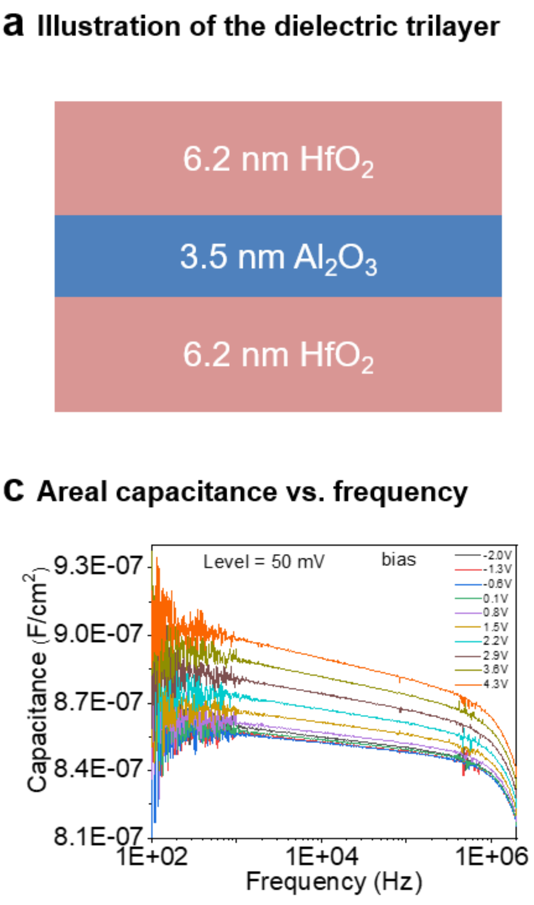

Figure S4. Structure and electrical characterization of the $HfO_2/Al_2O_3/HfO_2$ dielectric layer. a) Schematic illustration of the $HfO_2/Al_2O_3/HfO_2$ dielectric triple layers. The nominal thickness of each layer is marked out. b) Leakage current behavior, c) areal capacitance vs. frequency and d) areal capacitance vs. voltage at 1 kHz of the $HfO_2/Al_2O_3/HfO_2$ dielectric layer using a MIM device structure.

(2) Mapping of V_{th} and SS with a gate bias from -2 V to 5 V

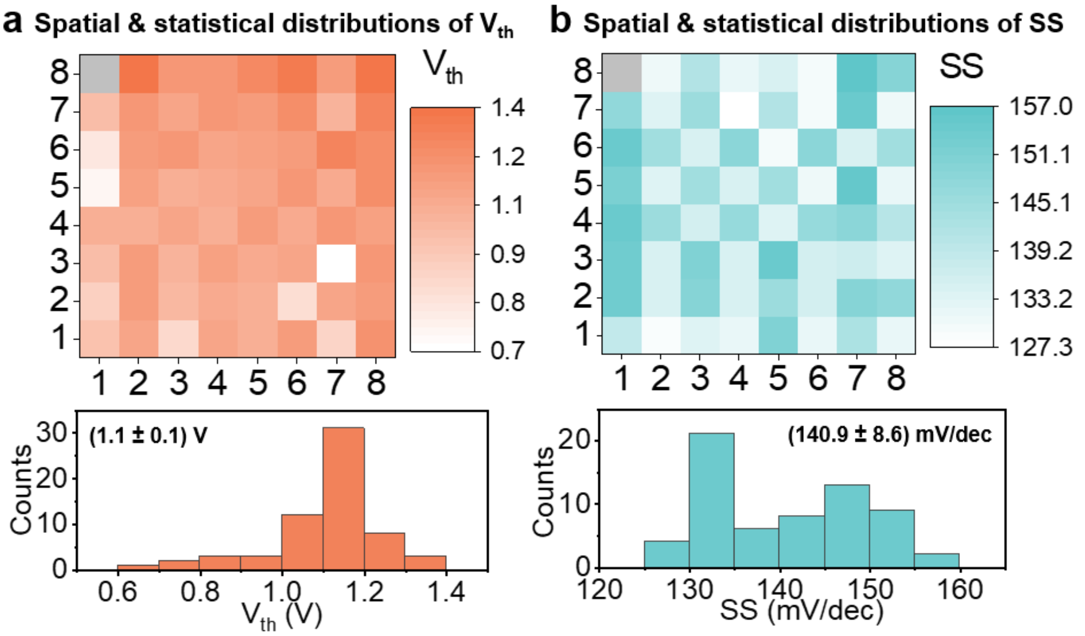

Figure S5. Mapping of V_{th} and SS with V_{GS} from -2 V to 5 V. a) Spatial and statistical distributions of V_{th}. b) Spatial and statistical distributions of SS. The average V_{th} is (1.1 ± 0.1) V and the average SS is (140.9 ± 8.6) mV/dec. The dysfunctional pixel is marked in the map by a gray block.

(3) Electrical performance of the a-IGZO TFTs with a low gate bias from -1 V to 1.8 V

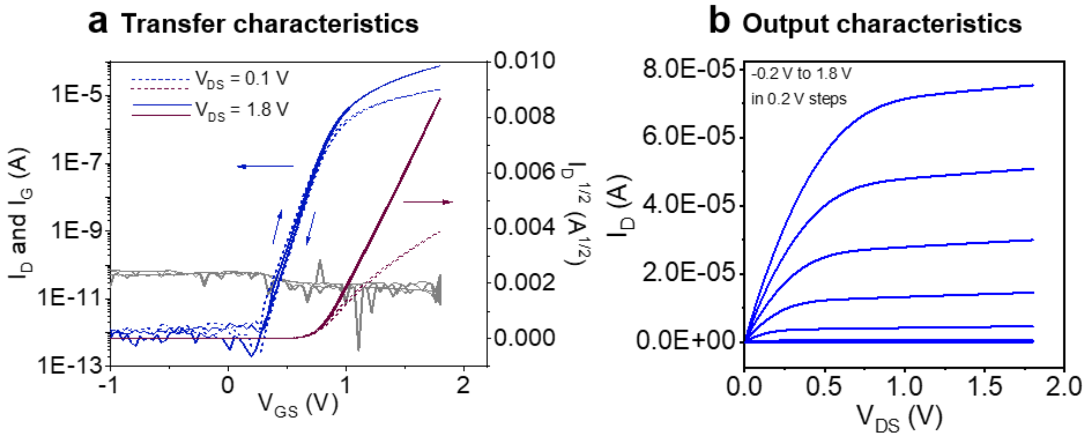

Figure S6. Electrical characterization of the a-IGZO TFTs with V_{GS} from -1 V to 1.8 V. a) Typical transfer characteristics at V_{GS} from -1 V to 1.8 V for the a-IGZO TFTs (L = 6.5 µm, W = 200 µm). The leakage current I_G is plotted with gray lines. b) Corresponding output characteristics at V_{GS} from -0.2 V to 1.8 V in 0.2 V steps.

(4) Electrical mapping of the a-IGZO TFT active-matrix with a gate bias from -1.8 V to 1.8 V

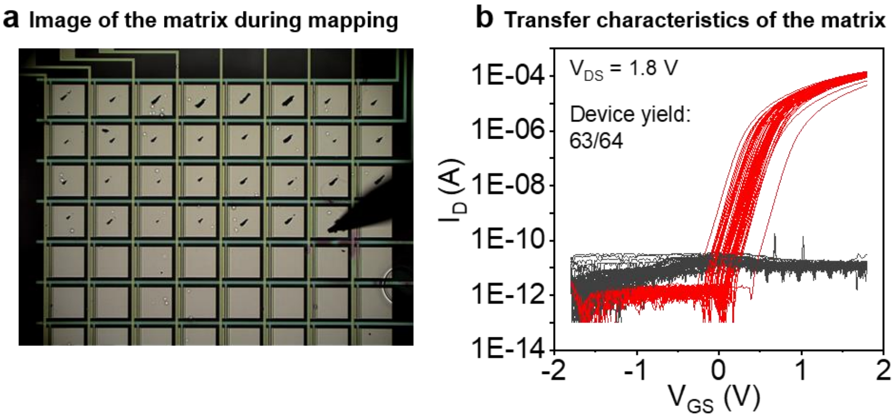

Figure S7. Electrical characterization of the a-IGZO TFT active-matrix with V_{GS} from -1.8 V to 1.8 V. a) Optical micrograph of the active-matrix during mapping. b) Transfer characteristics of all the 63 working TFTs in the 8 × 8 matrix. The leakage currents are plotted with gray lines.

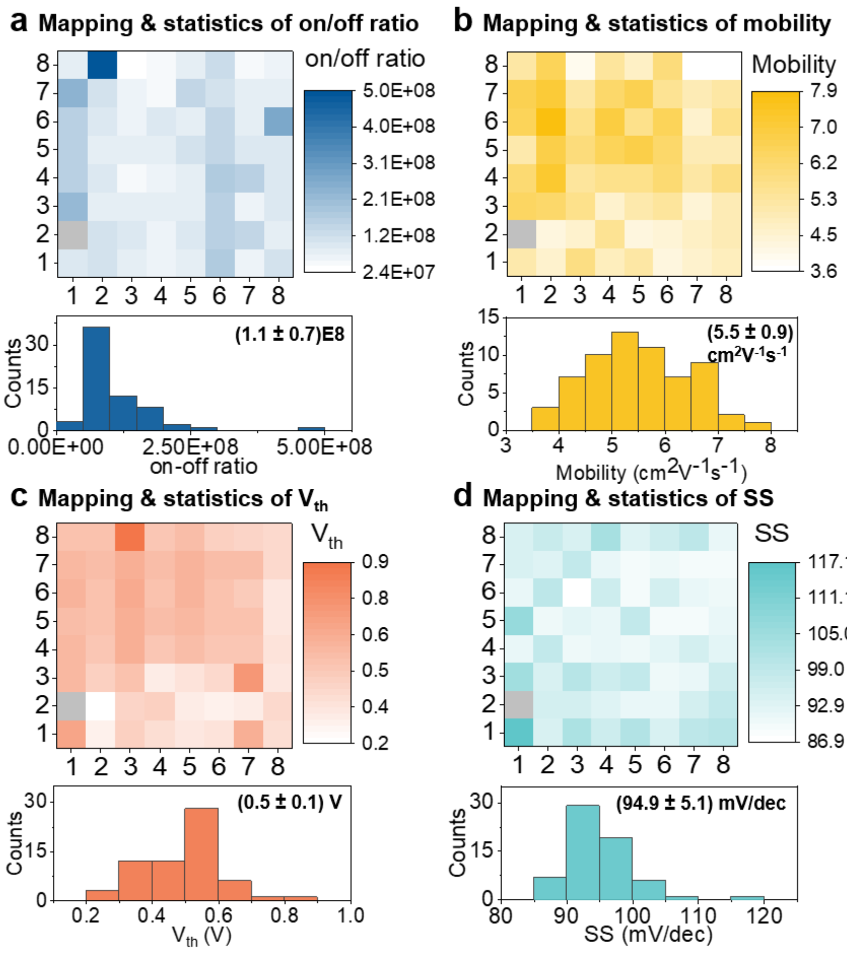

Figure S8. Spatial and statistical variations of the electrical properties of the a-IGZO TFTs in the 8 × 8 active-matrix mapping with V_{GS} from -1.8 V to 1.8 V. Spatial and statistical distributions of a) on/off ratio, b) mobility, c) V_{th} and d) SS. The average values are marked on the upper right in the corresponding histograms. The dysfunctional pixel is marked with a gray block.

(5) Environmental stability of the TFT electrical performance

Environmental stability test was carried out to check the long-term operation stability of our IGZO TFTs, as shown in Supplementary Figure S9. TFT testing structures (L = 6.5 µm, W = 150 µm) were immersed into DI water and kept at 85°C for a certain time. Then the sample was taken out and dried with nitrogen for measurement. The transfer characteristics of seven TFTs (T1 to T7 in Supplementary Figure S9a) on the sample were continuously monitored every 3 hours within the first 12 hours, and also recorded after 24 h. Typical transfer curve change over time is shown in Supplementary Figure S9b. We can see that a pronounced variation of the transfer characteristics occurs for the first 3 h, then the change is not significant any more. After 24 h in hot water the TFTs are still functioning well, as shown in Supplementary Figure S9c. It is surprising that all the seven TFTs under monitoring have survived the test although delamination of the SU-8 passivation layer is observed in some areas after 24 h (Supplementary Figure S9a). No delamination is present in the channel areas, probably due to the good adhesion of the SU-8 passivation layer to the channel regions. Thus, the channels are still intact during the test and this delamination has not affected the performance of the TFTs. The changes of the average parameters including on/off ratio, mobility, threshold voltage V_{th}, and subthreshold swing SS are shown in Supplementary Figure S9d and e. The mobility decreases constantly from (8.2 ± 0.8) $cm^2\,V^{-1}\,s^{-1}$ to (6.2 ± 1.4) $cm^2\,V^{-1}\,s^{-1}$. The on/off ratio drops significantly within the first 3 h and then stabilizes above 1×10^6. The V_{th} increases by 0.8 V within the first 3 h and then stabilizes at around 2.2 V. The SS value varies within 120 mV/dec to 140 mV/dec and does not show any clear change trend over time. From the environmental stability test we can conclude that our IGZO TFTs are very stable even in hot DI water. Additional passivation with inorganic layers could be employed to improve the delamination of SU-8 thus further increasing the device life time.

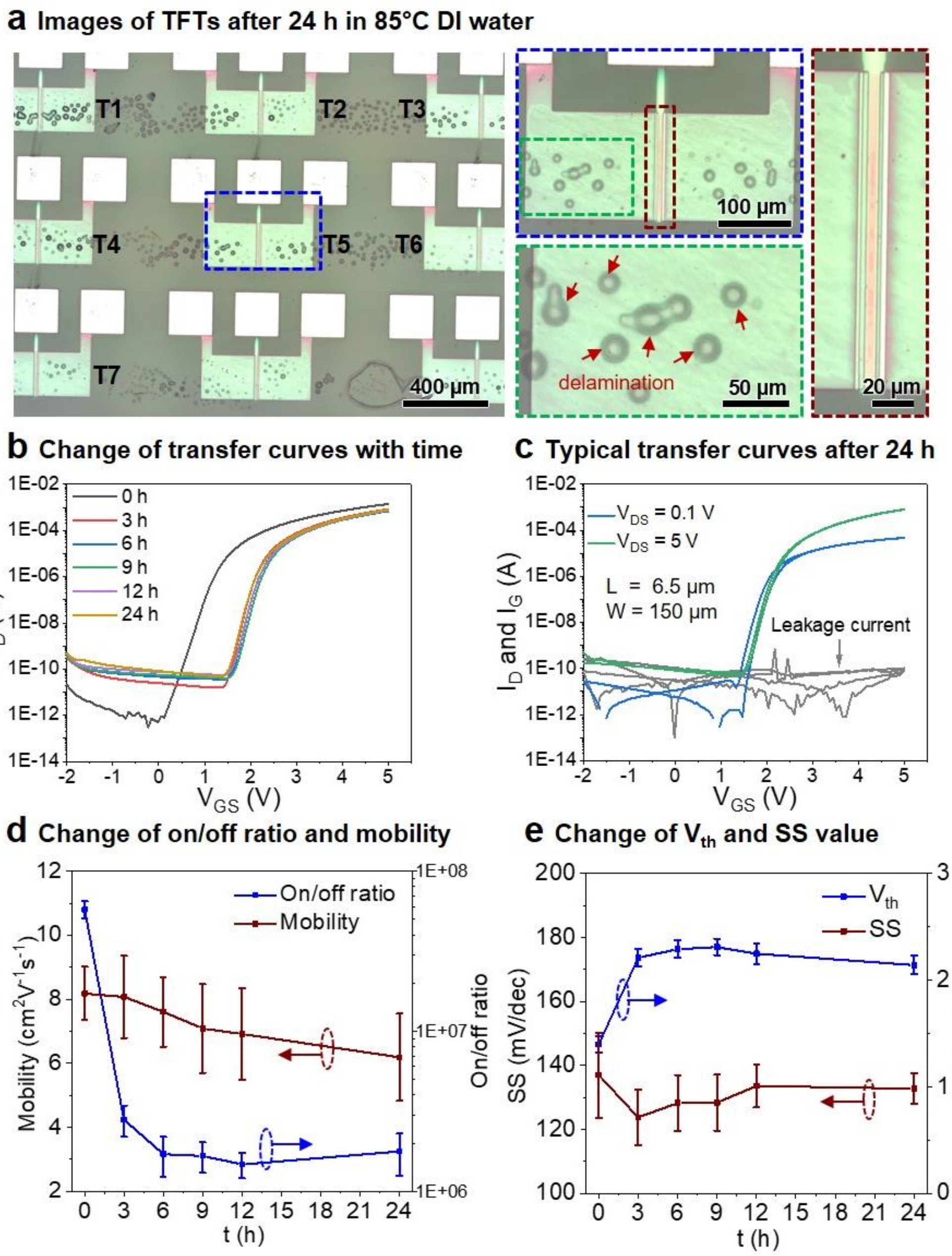

Figure S9. Environmental stability test for the IGZO TFTs. a) Images of the IGZO TFTs after immersed in 85°C DI water for 24 h. b) Typical transfer curve change over time. c) Typical transfer curves for TFTs after immersed in 85°C DI water for 24 h. d) Change of on/off ratio and mobility over time. e) Change of V_{th} and SS over time.

(6) Constant-voltage-bias-stress stability of the IGZO TFTs

A constant-voltage-bias-stress test was carried out to investigate the electrical stability of the IGZO TFTs as shown in Supplementary Figure S10. From Supplementary Figure S10a we can see that during the stress the drain currents drop fast at the beginning and then decrease gradually. The drain current decreases from 1.26 mA to 0.44 mA for V_{GS} = V_{DS} = 5 V, and

decreases from 0.062 mA to 0.019 mA for V_{GS} = 5 V, V_{DS} = 0.1 V. The leakage currents do not increase during the stress. The transfer curves before and after the stress are shown in Supplementary Figure S10b. The TFT is still functioning well after the stress process. The constant-voltage-bias stressing results in a positive transfer curve shift and a small decrease of the on-current. The shift of the cut-off voltage and decrease of the drain current are consistent with previously reported results.[6] The change of the transfer characteristics after the constant-voltage-bias-stress is probably due to the trapping of charges in the defects in the dielectric layer, the interface between the dielectric layer and the semiconductor, or within the semiconductor. This constant-voltage-bias-stress instability could be further improved in the future by optimizing the fabrication process, such as carrying out specific aging in water environment or/and post annealing, optimizing the ALD process for dielectric layers and the sputtering process for the semiconductor layer.

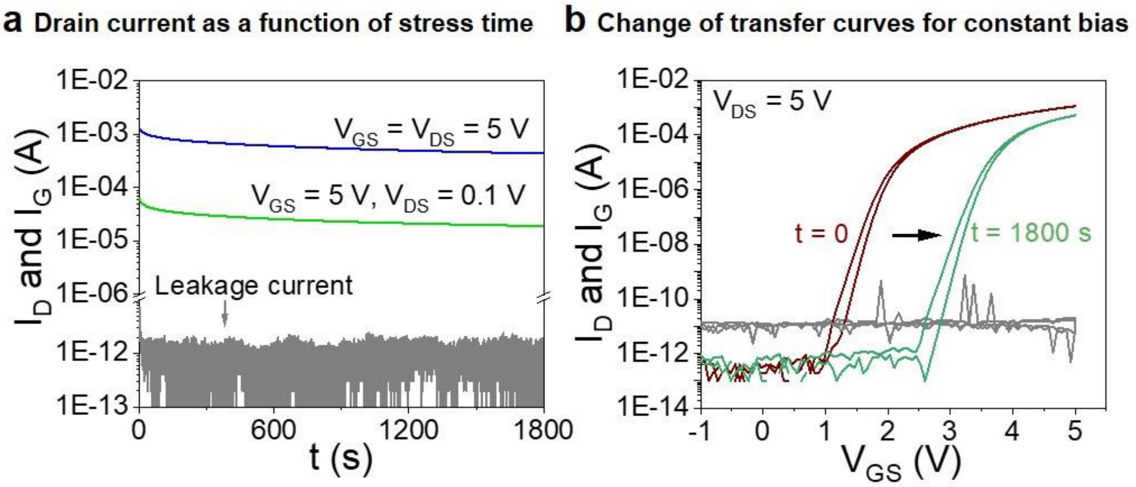

Figure S10. Constant-voltage-bias-stress test for the IGZO TFTs. a) Drain currents and gate leakage currents change as a function of stress time. The drain currents for V_{GS} = V_{DS} = 5 V and V_{GS} = 5 V, V_{DS} = 0.1 V are recorded respectively. The leakage currents are also shown. Over a stress duration of 1800 s the drain currents drop within one order of magnitude and the leakage currents do not increase. b) Transfer curves before and after the constant-voltage-bias-stress test over a duration of 1800 s. The constant-voltage-bias stressing results in a positive transfer curve shift and a small decrease of the on-current.

Supplementary Note 3 Digital electrochemistry of PPy

(1) Concept of the digital electrochemistry approach

Supplementary Figure S11a illustrates the concept evolution of the digital electrochemistry approach. Normally, two- or three-electrode configurations are used for electrochemical reactions.[7] For simplicity, here we plot a two-electrode system to demonstrate the concept. In the normal two-electrode configuration we connect a switch to the WE. The electrochemical reaction will take place when the switch is turned on and will stop when the switch is off. Therefore, the electrochemical reaction can be digitally controlled between on and off states by the switch. Since transistors can work as switches, we can replace the switch by a transistor and control the electrochemical reaction through a single transistor. Furthermore, we can integrate a transistor active-matrix onto the WE and perform electrochemical reactions on the pixel pads through digitally addressing the transistors within the active-matrix. The concept of digital electrochemistry can be broadened to other electrochemical reaction configurations.

(2) Digital electrochemical deposition of PPy on the a-IGZO TFT active-matrix

The deposition of PPy was performed in a pyrrole monomer aqueous solution containing 0.1 M pyrrole (Sigma-Aldrich Chemie GmbH, Taufkirchen, Germany) and 0.1 M NaDBS (Sigma-Aldrich Chemie GmbH, Taufkirchen, Germany). Both two- and three- electrode configurations were used in our experiments. Depositions on individual pixels were performed with the three-electrode configuration to observe the on-off operation behavior of TFTs in solution, the PPy growth I (t) curves, as well as the oxidation and reduction of the deposited PPy during CV scan. The three-electrode system was set up by configuring a two-channel Precision Source/Measure Unit (B2902A, Keithley, OR, USA), with one channel providing the gate bias and another channel providing the source bias. The a-IGZO TFT active-matrix functioned as the WE, and a sputtered 5 nm Ti/50 nm Au film on glass was used as the CE. Ag/AgCl was used as the reference electrode (RE).

A two-electrode system was used to carry out site-selective electrochemical deposition of PPy on multiple pixels. In this case, a microcontroller was used to facilitate the pixel selection. The a-IGZO TFT active-matrix was bonded to a PCB and connected to the output of the microcontroller. The active-matrix was used as the WE in the two-electrode system, and Ag/AgCl or Au was used as the CE and connected to the ground of the microcontroller. The microcontroller could be controlled through a customized Python interface to address specific pixels for the site-selective deposition. The output voltage through an 8-stage shift register in the gate lines was fixed as 5 V, and the output voltage in the source lines could be modulated from -2.4 V to 2.4 V through a digital-to-analogue-converter (DAC) voltage source. The deposition time could be set through the Python interface. The deposition process was in-situ recorded by a Leica camera. The scheme of the two-electrode system for multiple pixel deposition is illustrated in Supplementary Figure S11b. The side view and top view of the setup are shown in Supplementary Figure S11c and d respectively. A single active-matrix device bonded to a PCB is shown in Supplementary Figure 11e.

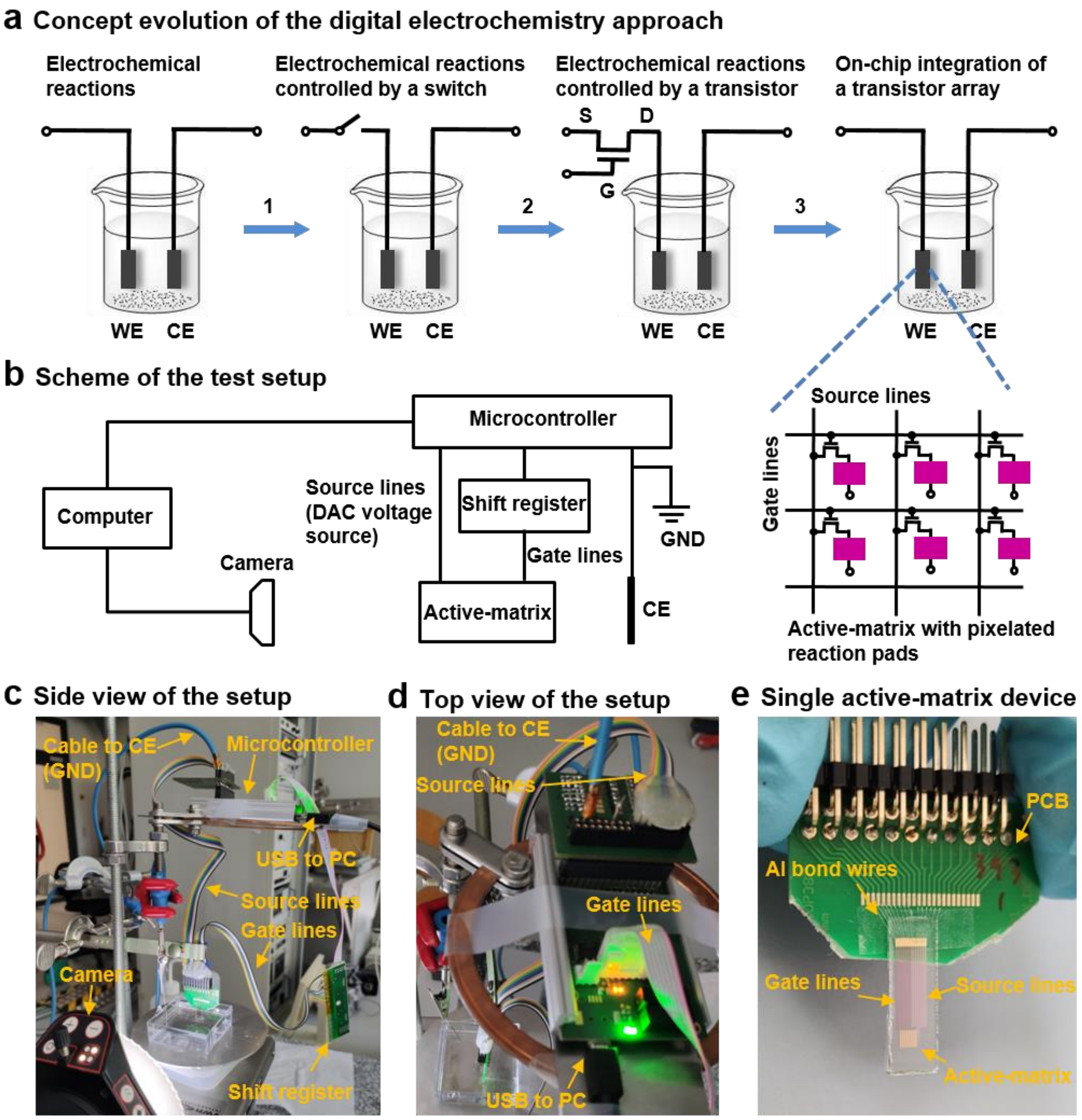

Figure S11. Concept of the digital electrochemistry approach and the test setup. a) Concept evolution of the digital electrochemistry approach. 1. Electrochemical reaction can be controlled by using a switch connected to the WE. 2. The switch can by replaced by a TFT. 3. Integrate a TFT active-matrix onto the WE and electrochemical reactions can take place on the pixel pads by digitally activating TFTs within the matrix. b) Scheme of the test setup. Photographs showing the c) side view and d) top view of the test setup. e, Photograph of a single active-matrix device bonded to a PCB.

(3) Morphological characterization of the PPy films

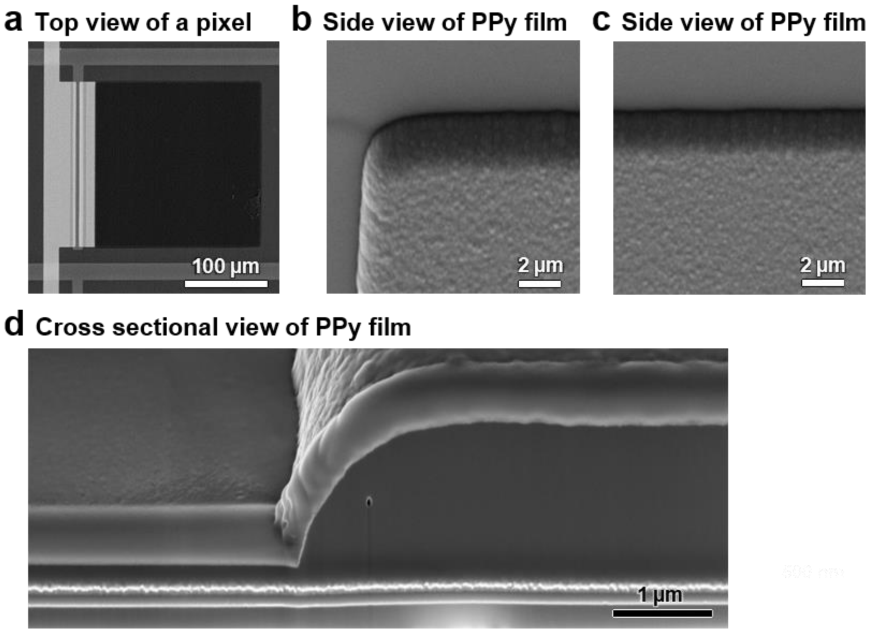

Figure S12. SEM characterization of the PPy film on a single pixel. a) Top view of a single pixel with PPy deposited (BSE contrast). b, c) 45° side views of the PPy film (SE contrast). d) FIB cross sectional view seen under 36°.

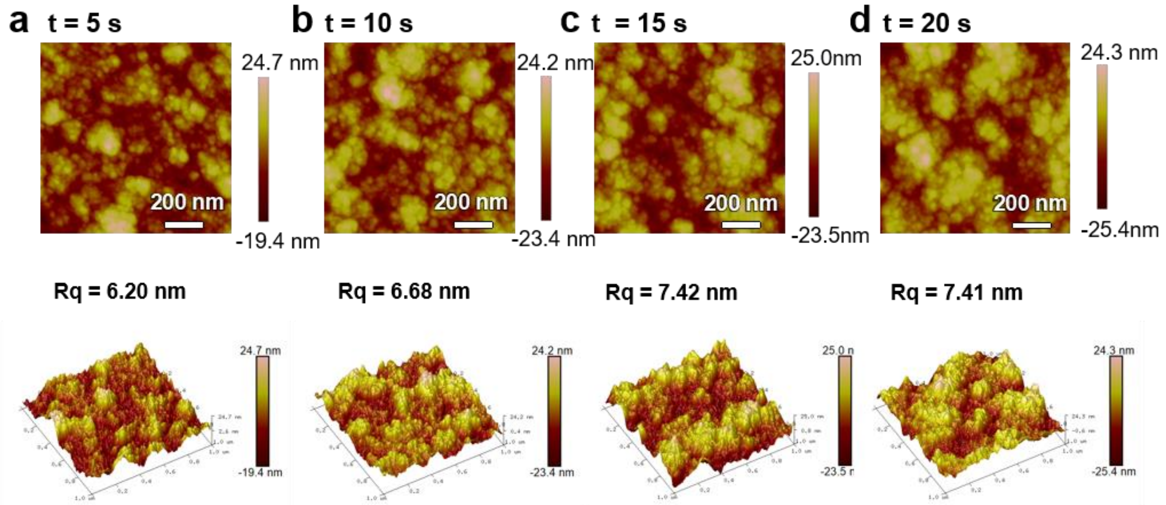

Figure S13. AFM characterization of the PPy films with different deposition time. The deposition time is a) 5 s, b) 10 s, c) 15 s and d) 20 s, respectively. The thickness of the PPy film in a-d) is 1.14 µm, 2.13 µm, 2.98 µm and 3.41 µm, respectively, from profilometer measurements. During the deposition the gate bias is fixed at 5 V, and source bias is set as 1.0 V vs. Au. The surface roughness is marked out in the corresponding images.

Supplementary Note 4 Digital electrochemical deposition and color switch of electrochromic materials

(1) Deposition and CV of PEDOT, P3MT and PDMA on individual pixels

Electrochemical depositions of PEDOT, P3MT and PDMA were first carried out respectively on individual pixels by using the three-electrode system as mentioned above. The deposition of PEDOT was performed in a propylene carbonate (PC, VWR GmbH, Darmstadt, Germany) solution containing 0.04 M 3, 4-ethylenedioxythiophene (EDOT, Sigma-Aldrich Chemie GmbH, Taufkirchen, Germany) and 0.1 M $LiClO_4$ (Sigma-Aldrich Chemie GmbH, Taufkirchen, Germany). The gate bias was kept as 5 V, and the source bias was set as 1.2 V vs. Ag/AgCl. The deposition time was 60 s. P3MT was deposited in a PC solution containing 0.1 M 3-methylthiophene (MeTh, Sigma-Aldrich Chemie GmbH, Taufkirchen, Germany) and 0.1 M $LiClO_4$. The gate bias was kept as 5 V, and the source bias was set as 1.9 V vs. Ag/AgCl. The deposition time was also 60 s. PDMA was deposited in a water-based solution containing 0.04 M 2, 5-dimethoxyaniline (DMA, Sigma-Aldrich Chemie GmbH, Taufkirchen, Germany) and 0.5 M H_2SO_4 (Alfa Aesar, MA, USA). The gate bias was kept as 5 V, and the source bias was set as 1.3 V vs. Ag/AgCl. The deposition time was 90 s. For all the depositions, the source-drain current I_S and leakage current I_G were recorded vs. time, as shown in Supplementary Figure 14a, d and g. The deposition currents for all the three materials are in the microampere range whereas the leakage currents are in the nanoampere range, indicating that the deposition could take place on specific pixels on the TFT active-matrix by gate and source bias controlling.

CV characterization was carried out after the material deposition. The CV of PEDOT and P3MT was performed in a PC solution containing 1.0 M $LiClO_4$. The gate bias was fixed as 5 V. The source bias was scanned from -1.4 V to 1.2 V vs. Ag/AgCl for PEDOT, and from -1.0 V to 2.2 V vs. Ag/AgCl for P3MT. The scan rate was controlled as 100 mV/s. The CV of PDMA was carried out in 0.5 M H_2SO_4 water-based solution. The gate bias was kept as 5 V, and the source

bias was scanned from -0.7 V to 1.0 V vs. Ag/AgCl with a rate of 100 mV/s. During the scans, I_S and I_G were recorded as shown in Supplementary Figure S14b, c, e, f, h and i. We can clearly see a pair of oxidation and reduction peaks for each material (Supplementary Figure S14b, e and h). The leakage current is as low as several nA in either PC solution or water-based solution and dose not increase during the 100 cycles of voltage scan (Supplementary Figure S14c, f and i), indicative of the high stability of the devices under aqueous and non-aqueous solutions.

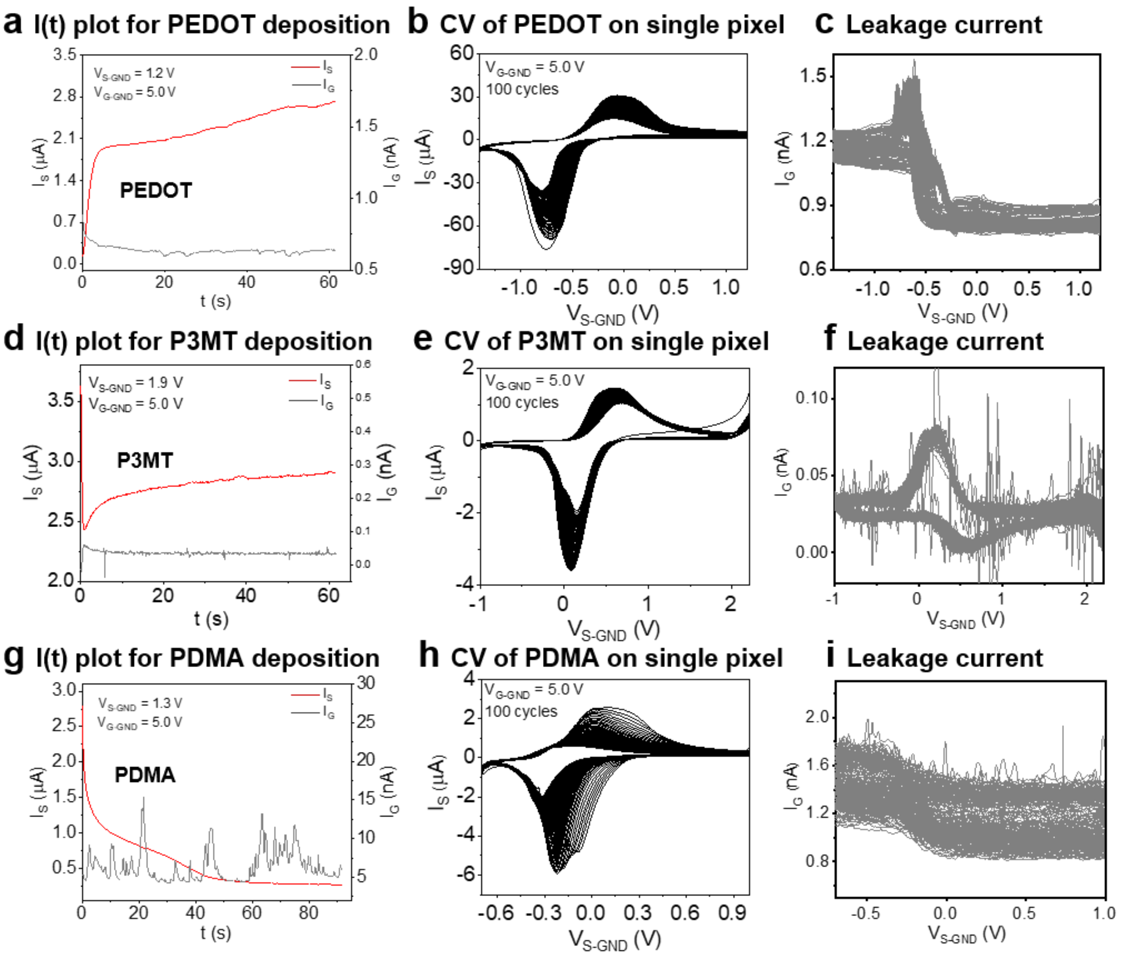

Figure S14. Deposition and CV of the electrochromic conducting polymers. Typical I (t) curves for the deposition of a) PEDOT, d) P3MT and g) PDMA on a single pixel. The gate bias $V_{G\text{-}GND}$ is 5 V and the source bias $V_{S\text{-}GND}$ is 1.2 V, 1.9 V and 1.3 V vs. Ag/AgCl respectively. CV of the deposited b) PEDOT, e) P3MT and h) PDMA on a single pixel. The $V_{G\text{-}GND}$ is 5 V for all the source bias scan. The corresponding leakage currents during the scan are plotted in c), f) and l) respectively.

(2) Deposition and electrochromic color switch of monochromatic patterns

The deposition of PEDOT, P3MT and PDMA monochromatic patterns was performed through the microcontroller in the two-electrode system, where the a-IGZO TFT active-matrix was used as the WE and Ag/AgCl was used as the CE. The other deposition parameters were the same with the deposition on individual pixels. Site-selective deposition took place on a specific pixel which was activated through the selection of the corresponding gate and source lines. Defined patterns could be deposited in a pixel-wise way, and deposition on multiple pixels at the same time could also be feasible by simultaneously activating multiple pixels.

The conducting polymers PEDOT, P3MT and PDMA demonstrated electrochromic color change under different redox states. The color change observation of PEDOT and P3MT was performed in PC solution containing 1.0 M $LiClO_4$, and the color change of PDMA was carried out in 0.5 M H_2SO_4 water-based solution. To change the color of a specific pixel, a fixed gate bias of 5 V was applied meanwhile a given source bias was applied to set the redox state of the electrochromic material. The conducting polymers on top of the pixel would be reversibly oxidized or reduced and displayed corresponding colors. The color change would take place on a single pixel or simultaneously on multiple pixels.

(3) Deposition and electrochromic color switch of multicolored patterns

Heterogeneous multicolored electrochromic patterns could be sequentially deposited in the monomer solutions. In our experiments, a colorful bird pattern composed of PEDOT body, P3MT beak and legs, and PDMA eye was deposited. Specifically, the PEDOT pattern was first deposited in the EDOT monomer solution. Then the sample was cleaned with PC solution and immersed into the MeTh monomer solution for the P3MT pattern deposition. Finally the sample was cleaned with PC and DI water, and immersed in the DMA monomer solution for the PDMA deposition. The color change observation of the electrochromic bird patterns was carried out in

0.5 M H_2SO_4 water-based solution. The applied source biases were the same with the single-material-composed monochromatic patterns.

Supplementary Note 5 Fabrication and actuation of the PPy actuator arrays

(1) Fabrication of active-matrix PPy actuator arrays

The PPy actuator arrays were fabricated based on the a-IGZO TFT active-matrix. Note that the reaction pads were removed from the S/D patterning mask (Supplementary Figure S15). Here a 100 nm Ti film was DC magnetron sputtered and dry-etched as the S/D layer. After the a-IGZO TFT active-matrix was fabricated, a lanthanum-acrylic acid-based organometallic photopatternable complex was spin-coated onto the sample with a thickness of 250 nm, and patterned to form a SL. The details of the synthesis and patterning of the SL were reported previously.[8] Then Au substrate layer (50 nm) was DC magnetron sputtered on top of the SL and wet-etched in the commercial Au etching solution. Subsequently, a SU-8 2000.5 passivation layer (400 nm) was spin-coated with a rate of 3700 rpm and patterned by photolithography. The completed sample was hard baked at 220°C for 10 min to completely cross link the SU-8, as well as increase the conductivity of the metal lines. Finally, PPy muscles were electrochemically deposited on the Au stripes and the SL was etched in 1.5 wt% HCl solution to obtain the freestanding PPy actuators.

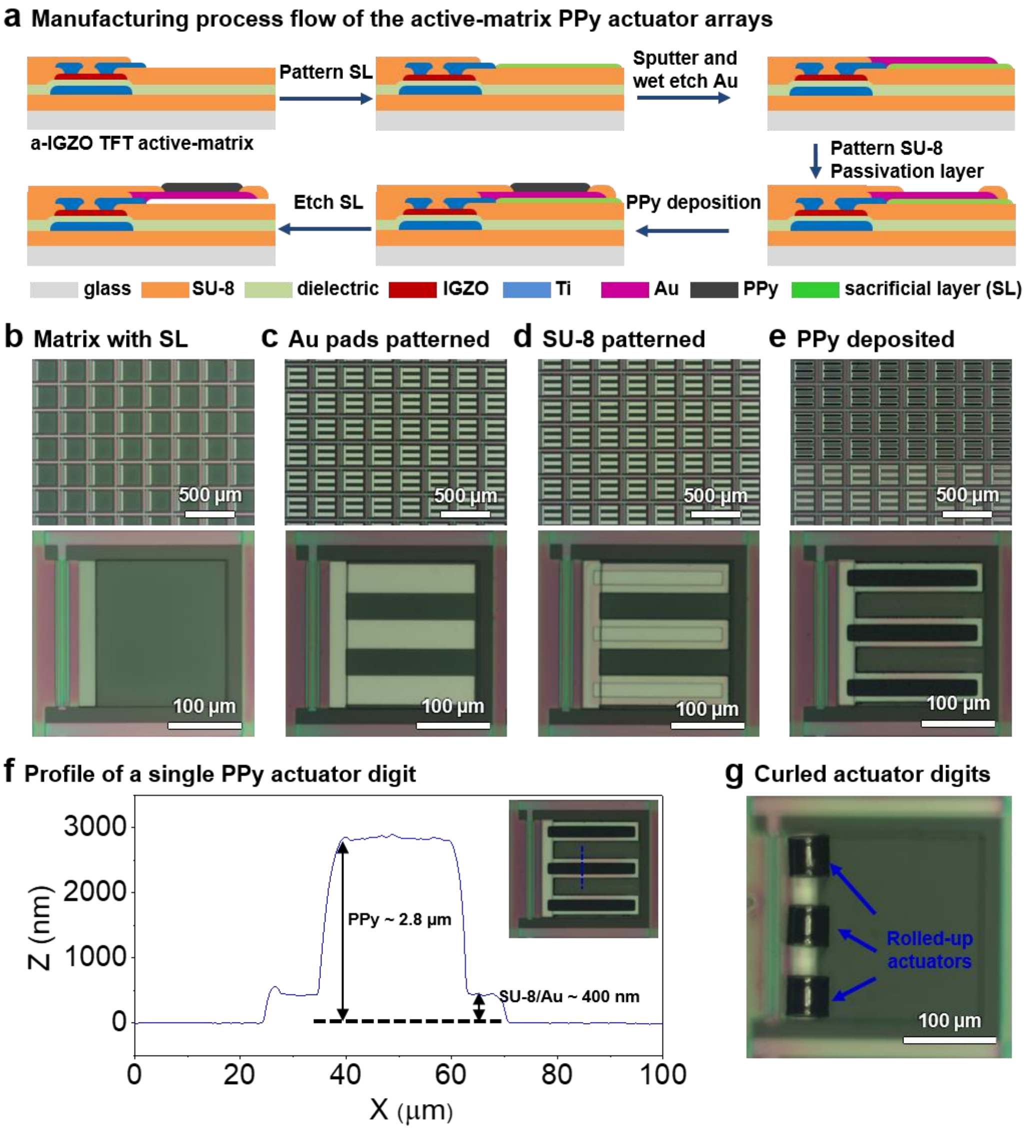

Figure S15. Fabrication and structure of the active-matrix PPy actuator arrays. a) Manufacturing process flow of the digital PPy actuator arrays. Optical micrographs of the a-IGZO TFT active-matrix and single pixel with b) a SL patterned on each pixel, c) Au substrate deposited and patterned, d) SU-8 passivation layer patterned and e) PPy electrochemically deposited. f) Profile of a single PPy actuator digit. g) Optical micrograph of one pixel with curled actuator digits.

(2) Programmable actuation of the PPy actuator arrays

Actuation of the PPy actuator arrays was carried out in 0.1 M NaDBS solution. To realize programmable actuation, scan of the source bias was carried out on those pixels addressed by the a-IGZO TFT active-matrix through the microcontroller. The source bias was scanned from 0.2 V to -1.3 V vs. Ag/AgCl with a rate of 100 mV/s. During the bias scan, the PPy was

reversibly oxidized and reduced. In the oxidation state, the PPy polymer chains would contract by repelling Na^+ and water into the solution, whereas in the reduction state the PPy polymer chains would swell by absorbing Na^+ and water from the solution. This volume change led to a periodic flattening and bending motion of the actuator digits. During the scan, the movement of the actuators was recorded by a Leica camera.

Supplementary References

[1] H. Yabuta, M. Sano, K. Abe, T. Aiba, T. Den, H. Kumomi, K. Nomura, T. Kamiya, H. Hosono, *Appl. Phys. Lett.* **2006**, *89*, 10.

[2] a) D. H. Cho, S. H. Yang, J. H. Shin, C. W. Byun, M. K. Ryu, J. I. Lee, C. S. Hwang, H. Y. Chu, *J. Korean Phys. Soc.* **2009**, *54*, 531; b) A. Kiazadeh, H. L.Gomes, P. Barquinha, J. Martins, A. Rovisco, J. V. Pinto, R. Martins, E. Fortunato, *Appl. Phys. Lett.* **2016**, *109*, 051606.

[3] W. Xu, H. Wang, L. Ye, J. Xu, *J. Mater. Chem. C.* **2014**, *2*, 5389.

[4] E. Yarali, H. Faber, E. Yengel, A. Seitkhan, K. Loganathan, G. T. Harrison, B. Adilbekova, Y. Lin, C. Ma, Y. Firdaus, T. D. Anthopoulos, *Adv. Electron. Mater.* **2020**, *6*, 1.

[5] M. M. Rahman, J. G. Kim, D. H. Kim, T. W. Kim, *Micromachines* **2019**, *10*, 1.

[6] K. Hoshino, D. Hong, H. Q. Chiang, and J. F. Wager, *IEEE Trans. Electron Devices* **2009**, *56*, 1365.

[7] F. Sassa, G. C. Biswas, H. Suzuki, *Lab Chip* **2020**, *20*, 1358.

[8] a) D. Karnaushenko, D. D. Karnaushenko, D. Makarov, S. Baunack, R. Schäfer, O. G. Schmidt, *Adv. Mater.* **2015**, *27*, 6582; b) D. Karnaushenko, N. Münzenrieder, D. D. Karnaushenko, B. Koch, A. K. Meyer, S. Baunack, L. Petti, G. Tröster, D. Makarov, O. G. Schmidt, *Adv. Mater.* **2015**, **27**, 6797; c) D. D. Karnaushenko, D. Karnaushenko, H. J. Grafe, V. Kataev, B. Büchner, O. G. Schmidt, *Adv. Electron. Mater.* **2018**, *4*, 1800298; d) C. Becker, D. Karnaushenko, T. Kang, D. D. Karnaushenko, M. Faghih, A. Mirhajivarzaneh, O. G. Schmidt, *Sci. Adv.* **2019** *5*, 1.
